# Reduced olfactory performance is associated with changed microbial diversity, oralization, and accumulation of dead biomaterial in the nasal olfactory area

**DOI:** 10.1128/spectrum.01549-23

**Published:** 2024-01-09

**Authors:** Christina Kumpitsch, Florian Ph. S. Fischmeister, Sonja Lackner, Sandra Holasek, Tobias Madl, Hansjörg Habisch, Axel Wolf, Veronika Schöpf, Christine Moissl-Eichinger

**Affiliations:** 1 Diagnostic and Research Institute of Hygiene, Microbiology and Environmental Medicine, Medical University of Graz, Graz, Austria; 2 Department of Psychology, University of Graz, Graz, Austria; 3 Department of Biomedical Imaging and Image-guided Therapy, Medical University of Vienna, Vienna, Austria; 4 BioTechMed, Graz, Austria; 5 Otto Loewi Research Center, Division of Immunology, Medical University of Graz, Graz, Austria; 6 Gottfried Schatz Research Center for Cell Signaling, Metabolism and Ageing, Molecular Biology and Biochemistry, Research Unit Integrative Structural Biology, Medical University of Graz, Graz, Austria; 7 Department of Otorhinolaryngology, Medical University of Graz, Graz, Austria; Johns Hopkins Medicine, Baltimore, Maryland, USA

**Keywords:** microbiome, olfaction, anosmia, nose, stool, dysosmia

## Abstract

The partial or complete loss of the sense of smell, which affects about 20% of the population, impairs the quality of life in many ways. Dysosmia and anosmia are mainly caused by aging, trauma, infections, or even neurodegenerative disease. Recently, the olfactory area—a site containing the olfactory receptor cells responsible for odor perception—was shown to harbor a complex microbiome that reflects the state of olfactory function. This initially observed correlation between microbiome composition and olfactory performance needed to be confirmed using a larger study cohort and additional analyses. A total of 120 participants (middle-aged, no neurodegenerative disease) were enrolled in the study to further analyze the microbial role in human olfactory function. Olfactory performance was assessed using the Sniffin’ Stick battery, and participants were grouped accordingly (normosmia: *n* = 93, dysosmia: *n* = 27). The olfactory microbiome was analyzed by 16S rRNA gene amplicon sequencing and supplemented by metatranscriptomics in a subset (Nose 2.0). Propidium monoazide (PMA) treatment was performed to distinguish between intact and non-intact microbiome components. The gastrointestinal microbiome of these participants was also characterized by amplicon sequencing and metabolomics and then correlated with food intake. Our results confirm that normosmics and dysosmics indeed possess a distinguishable olfactory microbiome. Alpha diversity (i.e., richness) was significantly increased in dysosmics, reflected by an increase in the number of specific taxa (e.g., *Rickettsia*, *Spiroplasma*, and *Brachybacterium*). Lower olfactory performance was associated with microbial signatures from the oral cavity and periodontitis (*Fusobacterium*, *Porphyromonas*, and *Selenomonas*). However, PMA treatment revealed a higher accumulation of dead microbial material in dysosmic subjects. The gastrointestinal microbiome partially overlapped with the nasal microbiome but did not show substantial variation with respect to olfactory performance, although the diet of dysosmic individuals was shifted toward a higher meat intake. Dysosmia is associated with a higher burden of dead microbial material in the olfactory area, indicating an impaired clearance mechanism. As the microbial community of dysosmics (hyposmics and anosmics) appears to be influenced by the oral microbiome, further studies should investigate the microbial oral-nasal interplay in individuals with partial or complete olfactory loss.

**IMPORTANCE:**

The loss of the sense of smell is an incisive event that is becoming increasingly common in today’s world due to infections such as COVID-19. Although this loss usually recovers a few weeks after infection, in some cases, it becomes permanent—why is yet to be answered. Since this condition often represents a psychological burden in the long term, there is a need for therapeutic approaches. However, treatment options are limited or even not existing. Understanding the role of the microbiome in the impairment of olfaction may enable the prediction of olfactory disorders and/or could serve as a possible target for therapeutic interventions.

## INTRODUCTION

The partial or complete loss of the sense of smell is a drastic event that substantially affects the quality of life by impacting psychological, behavioral, and social performance. Various studies have already reported its negative effects on personal hygiene, which can range from weak to extensive, but effects on social interaction and internal safety warning systems, for example, smoke, gas, or spoiled food, are also possible [reviewed in reference (1)]. In addition, the diet of those affected is often saltier and spicier, as the sense of smell is strongly linked to the sense of taste (2). The term “dysosmia” describes the condition of impaired olfactory function and combines the two conditions of “hyposmia” (reduced olfactory function) and “anosmia” (complete loss of olfactory function).

Dysosmia is mainly caused by various diseases, mechanical impacts, the aging process, or infection (3, 4). This loss of smell usually recovers spontaneously after an upper respiratory tract infection (URT) (5); however, it is not yet clear why dysosmia can even persist for years in some cases.

**Fig 1:**
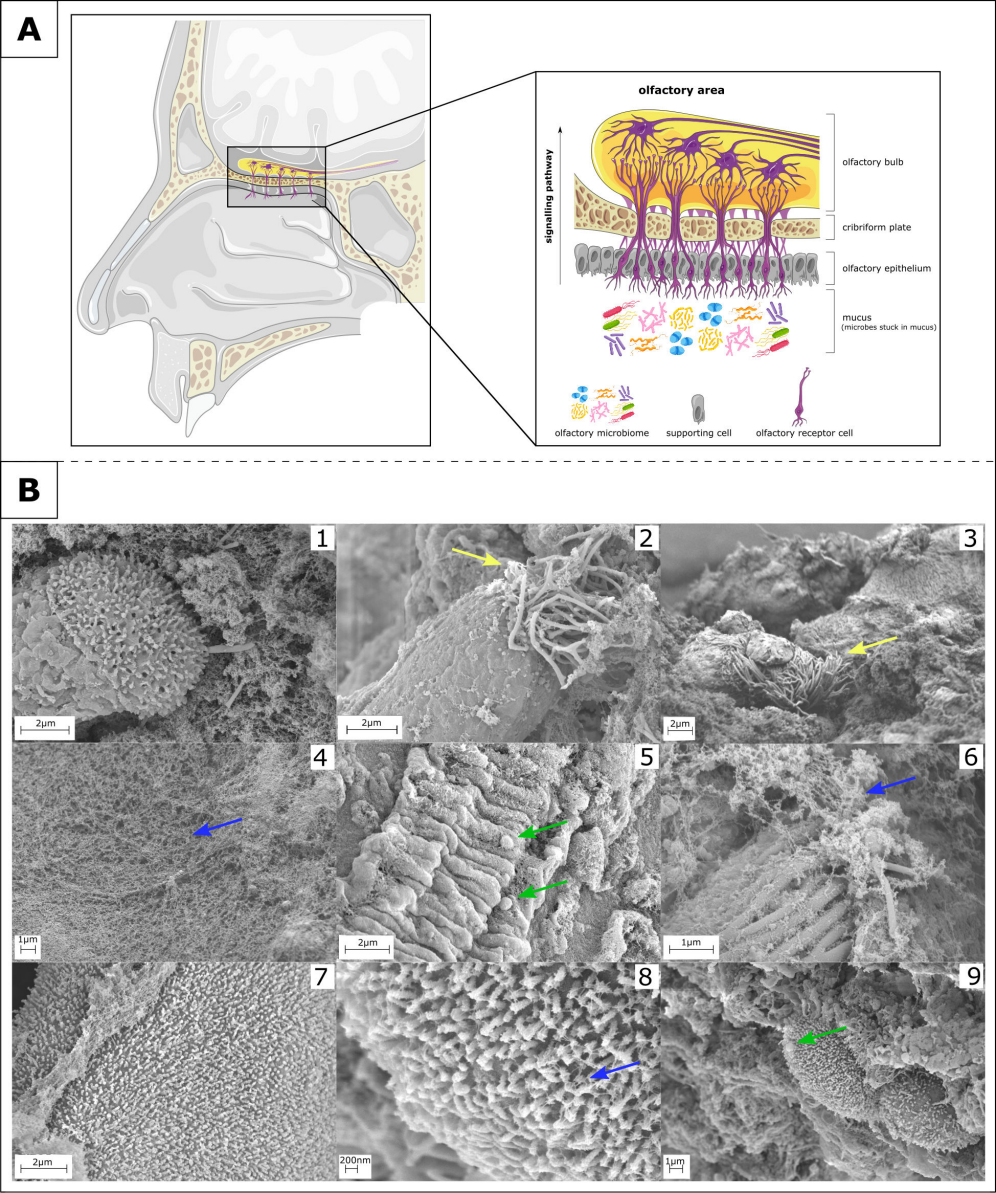
Olfactory mucosa—sampling area of interest located at the ceiling of the nasal cavity. (**A**) The olfactory microbiome is embedded in the mucus of the olfactory epithelium, including the olfactory receptor cells. The axons of these receptor cells reach through the cribriform plate into the olfactory bulb in the brain [image sources: nasal cavity and olfactory bulb—Servier Medical Art (19, 20), microbes: freepik (21); images modified with Inkscape (22)]. (**B**) Scanning electron micrographs (SEM) of a normosmic nasal sample: 1, a nasal epithelial cell; 2 a ciliated nasal epithelial cell; 3, cilia; 4, nasal phlegm/mucus; 5, bacteria-like structures; 6, nasal phlegm; 7, squamous epithelial lining; 8, squamous epithelial cell covered with mucus; 9, squamous nasal epithelial cells with bacteria-like structures; yellow arrow, cilia; green arrow, bacteria-like structures; blue arrow, nasal phlegm.

In general, olfactory performance is mediated by olfactory receptor cells in the ceiling of the nasal cavity—the olfactory mucosa. These cells extend into the brain with their axons passing through the cribriform plate into the olfactory bulb (Fig. 1A) (6–8). In addition to odor molecules, olfactory receptors are also able to recognize bacterial secondary metabolites (e.g., short-chain fatty acids and formyl-peptides). These molecules derived from bacteria can trigger the immune response mediated by the sense of smell. Thus, olfaction contributes to the individual’s defense against pathogens (9–11). The receptor neurons are surrounded and supported by sustentacular cells, and the entire area is covered with mucus (Fig. 1B) (8, 12). The mucosa of the olfactory epithelium is also colonized by numerous microorganisms, referred to as the olfactory microbiome (Fig. 1) (13–15). Interestingly, it has been shown that the human microbiome is able to produce volatiles (e.g., butyrate—strong unpleasant odor) which are sometimes perceived by the olfactory system and can even lead to the so-called “phantom odors” (16–18).

In a pilot study, our research group demonstrated that changes in the olfactory microbiome could be associated with altered olfactory performance. Significant differences in the microbiome diversity and composition were observed in hyposomics as compared with normosomics. Especially butyrate-producing bacteria (usually found in the gut) were significantly more abundant in hyposmics (13). In a study of olfaction and the sinus microbiome, Biswas et al., 2020, observed a loss of microbial diversity in dysosmics, noting that *Corynebacterium* (ASV46) and *Streptococcus* (ASV65) were significantly more abundant in normosmics, while *Streptococcus* (ASV111) and *Anaerococcus* (ASV43) were significantly more abundant in dysosmics (23).

Both studies (i.e., Koskinen et al., 2017 and Biswas et al., 2020) focus on the total detectable microbial community, which also includes dead microbial material (13, 23). However, 7,000 L of air from the external environment passes through the upper respiratory tract of a healthy person daily. Therefore, it is likely that among the 10^4^–10^6^ bacterial cells per m^3^ of inhaled air per day, inactive microbial material or external microbes may also be present that are not adapted to the habitat conditions. This material is usually transported by mucociliary clearance/ciliary beating towards the nasopharynx and down the throat (24–26).

This present study was conceptualized in order to deepen our current knowledge about the olfactory microbiome in normosmics and dysosmics. Guided by findings of our previous study (13), additional volunteers were recruited (*n* = 53) and subjected to olfactory performance testing, a more specific sampling of the olfactory mucosa only, and a thorough analysis of the bacterial and non-bacterial microbiome composition. We wanted to determine the potential influence of dead microbial material on olfactory function and discover whether this dead material originates from the gut. Furthermore, we were also interested in measuring the effect of a putative changed diet on the gastrointestinal microbiome, due to the reported changes in the diet of dysosmics.

Indeed, dysosmics tend to have a higher alpha diversity with a higher fraction of microbial material as compared with normosmics. However, the gut has not been identified as the source of these dead microbial signatures. Only *Methanobrevibacter* signatures belonging to a similar clade as fecal references have been found to be more prominent in dysosmics. In general, the diet of normosmics seems to be healthier than that of dysosmics.

## MATERIALS AND METHODS

### Design and subjects of the study

To replicate our pilot study (13), we recruited an additional 53 healthy volunteers aged 18–45 years. Great efforts were made to allow for comparability between the pilot study and Nose 2.0. Participants of both studies had to meet the following criteria: non-smoker, absence of psychiatric, neurological, or internal medicine conditions, use of probiotics and antibiotics within the month prior to sampling, no presence of nasal polyps, no use of nasal spray on the day of the study, and an absence of allergies (including acute hay fever or pollen) or acute illness (e.g., rhinitis). The only addition was the exclusion of participants with gastrointestinal disorders in Nose 2.0. Furthermore, no participant of the pilot study was included in the Nose 2.0 study. Because of the susceptibility of swab samples from the nasal olfactory region to contamination from other sites, we used sinus secretion collectors in Nose 2.0. While only 16S rRNA gene sequencing was performed in the pilot study for the microbiome analyses, the nose samples collected in Nose 2.0 underwent additional metagenomics and metatranscriptomics analyses. Furthermore, stool samples were collected for 16S rRNA gene-based amplicon sequencing and metabolomics. General metadata information (e.g., sex, age, and BMI) was collected in both studies (Table S1). In addition, the participants completed questionnaires in order to collect dietary as well as cognitive and emotional information (LEIDEN-R, SPM, and FAIR; no significant differences observed; data not included herein) and underwent olfactory testing (Sniffin’ Sticks Battery). For study schemata, see also Fig. S1.

### Olfactory testing and olfactory group definition

The olfactory function of the study participants was investigated using the Sniffin’ Sticks battery (Burghart Instruments, Wedel, Germany). Detailed information about this method can be found in reference (27). Briefly, a combined TDI score was defined by adding three different odor scores: the detection threshold (lowest odor concentration that can be detected), odor discrimination (ability to discriminate between odors), and odor identification (ability to name odors) (27).

The main categorization of participants was based on the measured TDI scores as follows (28): Normosmia (N) is defined by a TDI score of >29 – dysosmia (D; TDI ≤ 29). For a more detailed analysis, both normosmics and dysosmics were further divided into subcategories: good normosmics (G; TDI ≥ 36.0), weak normosmics (W; 36.0 > TDI > 29.0), hyposmics (H; 29.0 ≥ TDI > 16.5), and anosmics (A; TDI ≤ 16.5).

### Dietary questionnaires

All participants had to complete the “German Food Frequency Questionnaires”' (Robert Koch Institute (29), to evaluate nutritional intake information. The responses were analyzed using the Austrian specific nutrition software (30). Values of nutritional intake were normalized based on the kcal intake (nutritional value per 100 kcal food intake) before being correlated with the olfactory performance measures (TDI scores and olfactory groups; Table S14) and the microbiome (nose and stool).

### Microbial sample collection and preparation

#### Nasal samples

Samples were collected by ENT physicians at the University Department of Otolaryngology, Medical University of Graz, Austria, using a sinus secretion collector (Medtronic Xomed Inc.). Samples were immediately placed on ice until further processing. Nasal samples were dissolved in 500 µL of an aqueous 0.9% (wt/vol) NaCl solution and divided between two tubes. One aliquot (250 µL) was treated with propidium monoacid (PMA). PMA is used to mask freely accessible DNA for subsequent PCR reactions and thus allows one to gain a reliable insight into the intact/viable fraction of microbiomes (31). The PMA treatment was performed in the dark. Samples were mixed with the PMA solution (final concentration: 50 µM), shaken briefly, and then incubated on a shaker for 10 minutes in a PMA-Lite LED photolysis instrument (Biotum). Subsequently, both aliquots (PMA treated and untreated) were stored at −20°C.

#### Stool samples

The stool samples were collected by the participants themselves on the same day that the nasal samples were collected. Samples were stored on ice until further processing. Within a maximum of 2 h, the samples were aliquoted: 0.1 g of stool was dissolved in 1 mL of an aqueous 0.9% NaCl solution and an aliquot of 500 µL was treated with PMA solution as previously described. Again, both aliquots (PMA treated and untreated) were stored at −20°C.

### DNA and RNA extraction

DNA from the nasal (PMA-treated and untreated) and stool (PMA-treated) samples was extracted using the DNeasy PowerSoil Kit (QIAGEN, USA). Untreated nasal samples which were frozen in liquid nitrogen immediately after sample collection were used for RNA extraction (RNeasy RNA Extraction Kit, QIAGEN, USA). DNA and RNA extractions were performed according to the manufacturer’s protocol with minor modifications: instead of vortexing the samples, a MagNaLyser was used at 6,500 rpm for two cycles of 30 seconds. The nucleic acid concentration obtained was quantified using the Qubit dsDNA HS Assay Kit (Thermo Fisher Scientific, USA) before samples were stored at −20 (DNA) and −80°C (RNA).

### Amplicon sequencing

#### 16S rRNA gene and ITS region-based amplicon sequencing

Three different approaches were used for amplicon sequencing: the universal (primer pair: 515F-806R) and the archaeal approaches (nested PCR: 344F-1041R, 519F-806R) were used to amplify the V4 region of the 16S rRNA gene and the fungal approach (primer pair: ITS86F-TS4R) was used to amplify the ITS region. PCR was performed on DNA extracted from stool samples using all approaches, whereas only the universal approach was used to amplify DNA extracted from nasal samples [see (32, 33) for detailed information on protocol and primers]. PCR products were sequenced using the Illumina MiSeq sequencing platform (Illumina, Eindhoven, The Netherlands) at the Core Facility for Molecular Biology of the Center for Medical Research in Graz, Austria (34).

#### Sequence data procession and control

Raw sequences (paired-end reads) were quality filtered and processed using QIIME 2 (Quantitative Insights Into Microbial Ecology) v2020.8. DADA2 (Divisive Amplicon Denoising Algorithm) in QIIME 2 was used to denoise the data before sequences were annotated using the reference databases SILVA v138 (universal approach), SILVA v138 (archaeal approach), and Unite v8.3 (fungal approach) as a Naïve Bayes classifier (detailed information can also be found here: Supplemental text) (35–39). After data processing, the R package decontam with default settings, a threshold of 0.5, and the prevalence method were used to treat extraction blanks and PCR negative controls (40) (https://github.com/benjjneb/decontam). In addition to the controls, mitochondrial signatures, unassigned sequences (and also human mitochondrial assigned ones), and features with zero or only one read were removed.

SRS normalization (SRS = scaling with ranked subsampling) (41) was then applied to the nasal samples with sample depths of 500 and 1006 (PMA-treated and untreated, respectively) and to the stool samples (bacterial approach only) with a cut-off at 2,222 read counts. The archaeal and fungal approaches provided read counts that were too low for some samples; therefore, these were not normalized. The remaining ASVs (Data set S1–S5) were visualized using various tools [including Microbiome Explorer v1.6.0 (42), Rstudio v4.1.2 (43), and RawGraphs (44)]. The colors and legends of charts and graphs were adjusted using Inkscape (22).

Non-normalized data were used to perform differential abundance analysis (e.g., LEfSe and ALDEx2) (45–47).

### Metatranscriptomics analysis

#### Metatranscriptome sequencing

RNA (without PMA treatment) extracted from 12 samples (6 normosmics and 6 anosmics) was sent to a company (Macrogen, South Korea) for metatranscriptomics sequencing. The TruSeq Nano DNA Construction Kit (Illumina, Eindhoven, The Netherlands) was used to construct the library before sequencing with the Illumina NovaSeq 6000 technique (Illumina, Eindhoven, The Netherlands).

#### Metatranscriptomics data processing

The raw data were analyzed using the metagenomics pipeline as previously described (37). Briefly, the quality of the sequences was checked using fastqc (v0.11.8) (48) before trimming (v0.38) (49), and the information derived from the human host (chromosomes hg19 and GChr) was removed by binning using bowtie2 (v2.3.5) (50). The remaining reads were analyzed by using a gene-centric [protein-based annotation using diamond (protein-based) and visualized with MEGAN (51)] and a genome-centric method [Megahit v1.1.3 (52), MaxBin v2.2.4 (53), and dRep v2.0.5 (54)].

Results from applying the gene-centered approach were filtered by removing all traits with fewer than three reads. To examine changes in all life domains, the following additional filters were set: filtering all (i) non-bacterial, (ii) non-archaeal, (iii) non-fungal, or (iv) non-viral signatures (Data set S6 to S9).

### Metabolomic analysis

Metabolomic measurements of PMA-untreated stool samples were performed at the Gottfried Schatz Research Center for Cell Signaling, Metabolism and Aging, Molecular Biology and Biochemistry, Medical University of Graz. A subset of 49 stool samples of the Nose 2.0 study was analyzed using untargeted NMR (nuclear magnetic resonance) spectroscopy for several metabolites in-house. Metabolites were extracted using a methanol/water solution and prepared for NMR measurements as described previously (55). NMR was performed on an AVANCE Neo Bruker Ultrashield 600-MHz spectrometer equipped with a TXI probe head at 310 K. Briefly, the 1D CPMG (Carr-Purcell-Meiboom-Gill) pulse sequence (cpmgpr1d, 512 scans, 73,728 points in F1, 11,904.76 Hz spectral width, 512 transients, and recycle delays of 4 s) with water suppression by pre-saturation was used for ^1^H 1D NMR experiments. Bruker Topspin version 4.0.2 was used for NMR data acquisition. Spectra for all samples were automatically processed (exponential line broadening of 0.3 Hz), phased, and referenced using TSP at 0.0 ppm, using the Bruker Topspin 4.0.2 software (Bruker GmbH, Rheinstetten, Germany). Spectra were imported to Matlab2014b, and the regions around the water, TSP, and remaining methanol signals were excluded. To correct for the sample metabolite dilution, a probabilistic quotient normalization step was performed. Metabolite quantification was based on the signal integration of normalized spectra as described previously (56).

### BioEnv

Using R Studio v4.1.2 (43) and its companion package (vegan (57), BioEnv plots were generated with olfactory performance, sex, age, BMI, and food intake variables, showing the positive or negative correlations (Spearman’s rho) with microbial community dissimilarities.

### Statistical analysis

IBM SPSS Statistics v26 (58) was used to perform statistical analyses (*P*-values). Parameters were tested for normal distribution before selecting the appropriate statistical test. In general, uncorrected significant values are reported as *P*-values (*P* < 0.05) and Bonferroni-corrected values as *q*-values (*q* < 0.05) in the manuscript.

### Correlation heatmaps

Rstudio v4.1.2 (43) with the following R packages was used to create the correlation heatmaps: ggpubr v0.4.0 (59), tidyverse v1.3.2 (60), textshape v1.7.3 (61), dplyr v1.0.9 (62), tibble v3.1.6 (63), and ggplot2 v3.3.5 (64). Since olfactory values are non-parametric [using SPSS (58)], the Spearman’s rho correlation was used.

### Source tracking

Source tracking was performed using sourceTracker2 (https://github.com/biota/sourcetracker2). The original version of SourceTracker was described in Knights et al. (65). For source tracking, only the data sets from Nose 2.0 were used (reads from PMA-treated stool samples and non-PMA-treated nasal samples from both olfactory groups). Reads from respective data sets were co-processed with QIIME 2 as described above. Stool samples were assigned as the “source,” whereas nasal samples served as the “sink.” The selected rarefaction depth for source tracking was 480 (for both sink and source), to allow all samples to be kept in the analysis.

### *Methanobrevibacter* tree

For the tree, all *Methanobrevibacter* sequences from the nasal samples (Nose 2.0) were used. Additional sequences (e.g., representatives of *M. oralis* and *M. smithii*), as well as environmental sources (rumen), were retrieved from SILVA (38). Environmental sequences were used to expand the tree. All archaeal sequences were subjected to the Silva Alignment, Classification, and Tree Service Tool (ACT (38)). The tree was computed using the “Compute tree” function in SILVA ACT with standard settings (no positional variability filter). The tree was then annotated using iTOL (66), and legends were added using Inkscape (22).

## RESULTS

### Description of the study and the study cohorts

A new study (Nose 2.0) was conducted to add to the results of our published pilot study (13); Fig. S1) which indicated the existence of a potential correlation between the nasal microbiome composition and olfactory function. In the following, the new study and the pilot study are referred to as “Nose 2.0” and “pilot,” respectively. In the new study, we recruited an additional 53 participants (22 women) and analyzed samples collected from these participants together with the already-collected 67 nasal samples (50 women) from the pilot study.

Based on the olfactory testing outcomes, the participants were categorized into two major groups, namely, normosmics (N: *n* = 33; TDI > 29) and dysosmics (D: *n* = 20; TDI ≤ 29), and four subgroups (good normosmics (G: *n* = 16; TDI ≥ 36.0), weak normosmics (W: *n* = 17; 36.0 > TDI > 29.0), hyposmics (H: *n* = 7; 29.0 ≥ TDI > 16.5), and anosmics (A: *n* = 13; TDI ≤ 16.5) (Fig. S2; Table S2). According to these cut-offs, participant groups from the pilot study were reclassified based on their olfactory performance. Only seven (D; N: *n* = 60) subjects met the criteria for olfactory dysfunction after adjustment (G: *n* = 24; W: *n* = 35; H: *n* = 8) (see also Fig. S2).

The observed different olfactory performance in both cohorts could not be explained by any observed metadata, such as age, BMI, or sex (Table S2). The homogeneity of the cohorts was further supported by the results of analyses of the metadata information based on the olfactory groups (major and subgroups) (Table S3).

Participants with dysosmia were selected based on the loss of smell they had experienced due to an infection that had occurred at least 1 year earlier. Based on inflammatory markers in the blood, no active inflammation was detected in any subject (Table S1). Notably, samples were collected before the COVID-19 pandemic—from April 2018 to November 2019.

### Diversity of nasal microbial community differs based on olfactory performance

To add to the collection of already-published data (13), the raw sequencing data from the pilot study had to be re-analyzed together with the Nose 2.0 data before combining/comparing the data sets (see Material and Methods). From both studies (combined data of both studies), an overall of 2,480 unique microbial features (120,720 reads) were obtained by 16S rRNA gene sequencing the “olfactory microbiome.” Notably, archaeal information made up approximately 2% of the data set (see data set 1).

**Fig 2:**
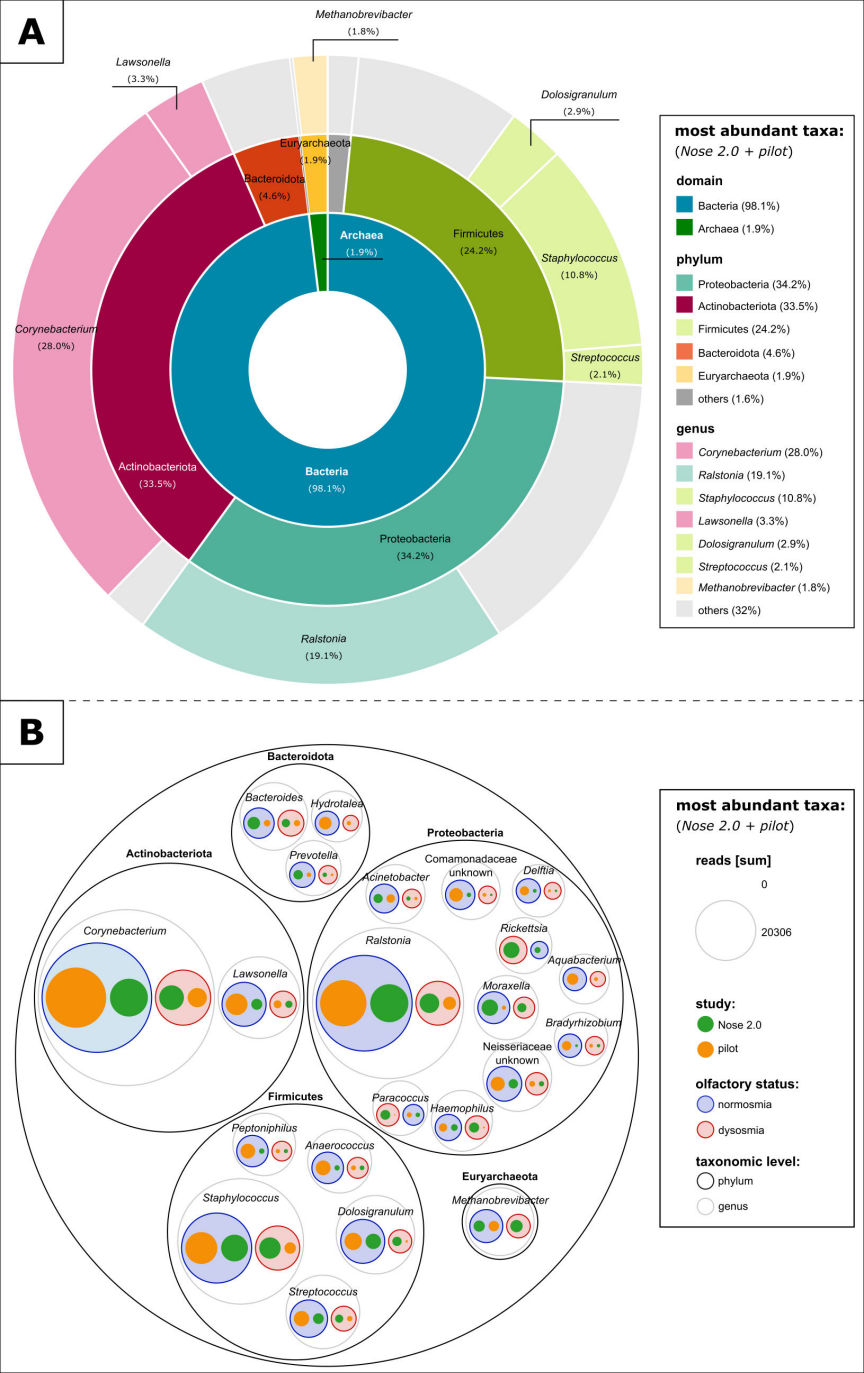
Relative abundance of the most abundant taxa found in the study cohorts. The plots show the 5 and 15 most abundant phyla and genera, respectively, (**A**) shared in both studies and (B) in the Nose 2.0 and pilot studies. Information on the olfactory performance is based on the main groups. Circle sizes indicate the number of reads of the specific microbial taxa found in the different groups.

The predominant taxonomic signatures and microbial composition were similar in both studies, with Proteobacteria, Actinobacteriota, Firmicutes, Bacteroidota, and Euryarchaeota representing the predominant phyla and *Corynebacterium*, *Ralstonia*, *Staphylococcus*, *Lawsonella*, and *Dolosigranulum* representing the most abundant genera (Table S4; Fig. 2A). However, only 243 features were shared between the studies, while 1,529 and 708 ASVs (amplicon sequence variants) were found to be unique for the pilot and Nose 2.0 data sets, respectively (Fig. 2B; Fig. S3; detailed list in Table S5).

**Fig 3:**
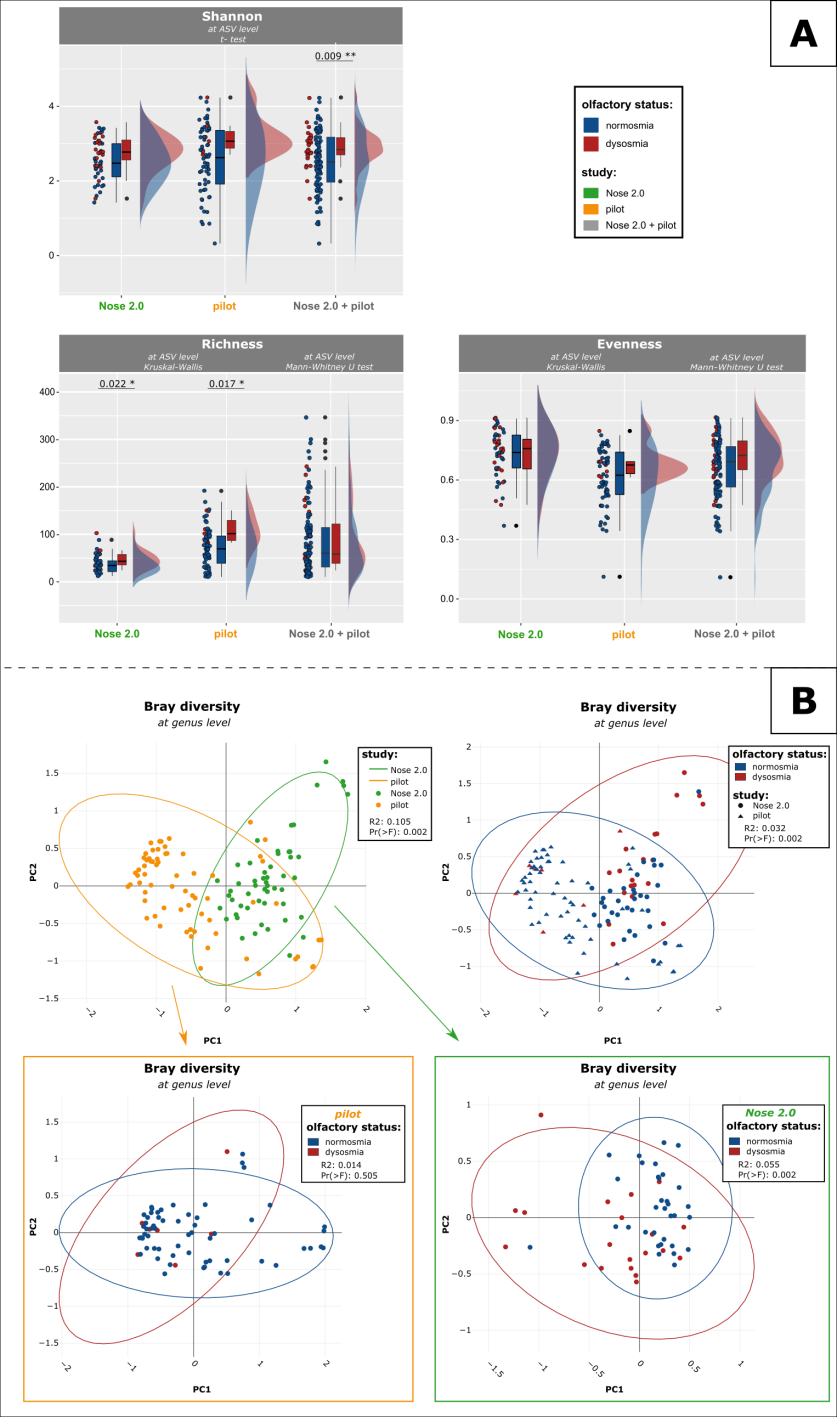
Microbial diversity differs between olfactory groups in both cohorts*.* (A) Higher alpha diversity (Shannon index) in dysosmic samples is driven by richness (Chao1) rather than evenness (*P*-values not corrected). (**B**) Clusters of nasal samples of normosmics and dysosmics overlap in a PCA plot (based on TDI).

Consistent trends in the alpha diversity of both cohorts (Nose 2.0 and pilot) were observed. The increased Shannon index of the dysosmics in both cohorts (compared to normosmics) was driven by the evenness; however, these differences were not significant. The increase in microbial richness (Chao1 index) based on the TDI score was significant. This result was supported by the trend observed in the Shannon index (Fig. 3A; *P*_Nose 2.0_TDI_richness_ = 0.022; *P*_pilot_TDI_richness_ = 0.017; Mann-Whitney U).

The differences observed in alpha diversity were even more pronounced if the main groups were further divided into olfactory subgroups (good and weak normosmia, hyposmia, and anosmia), showing a gradual/stepwise increase as the olfactory perception decreased; again, this result may have been mainly driven by the data richness (Chao1) rather than evenness (Fig. S4; not significant).

The microbial community information indicated a partial overlap of the clusters of both cohorts in a PCA plot (not significant). The Nose 2.0 cohort revealed a significant difference between normosmics and dysosmics; however, this was not reflected in the pilot cohort (Fig. 3B; *P*_NBA_ = 0.002; *P*_pilot_ = 0.505 TDI; Adonis test).

Both studies revealed a greater variability in the dysosmic microbiomes than in the normosmic microbiomes, revealed by the significant difference between these olfactory groups and subgroups seen in Nose 2.0 and the pilot study, respectively [Fig. 3B; red dots; *P*_Nose2.0 + pilot_ = .002, *R*^2^_Nose2.0 + pilot_ = .032; *P*_Nose2.0_ = 0.008, *R*^2^_Nose2.0_ = 0.055; *P*_pilot_ = 0.627, and *R*^2^_pilot_ = 0.014 (Fig. S4)].

Many taxa were identified that are unique to the microbiomes of either normosmics or dysosmics. Although both studies revealed slight differences with respect to the indicative microbial signatures, several taxa were identified as potential signatures for olfactory performance.

**Fig 4:**
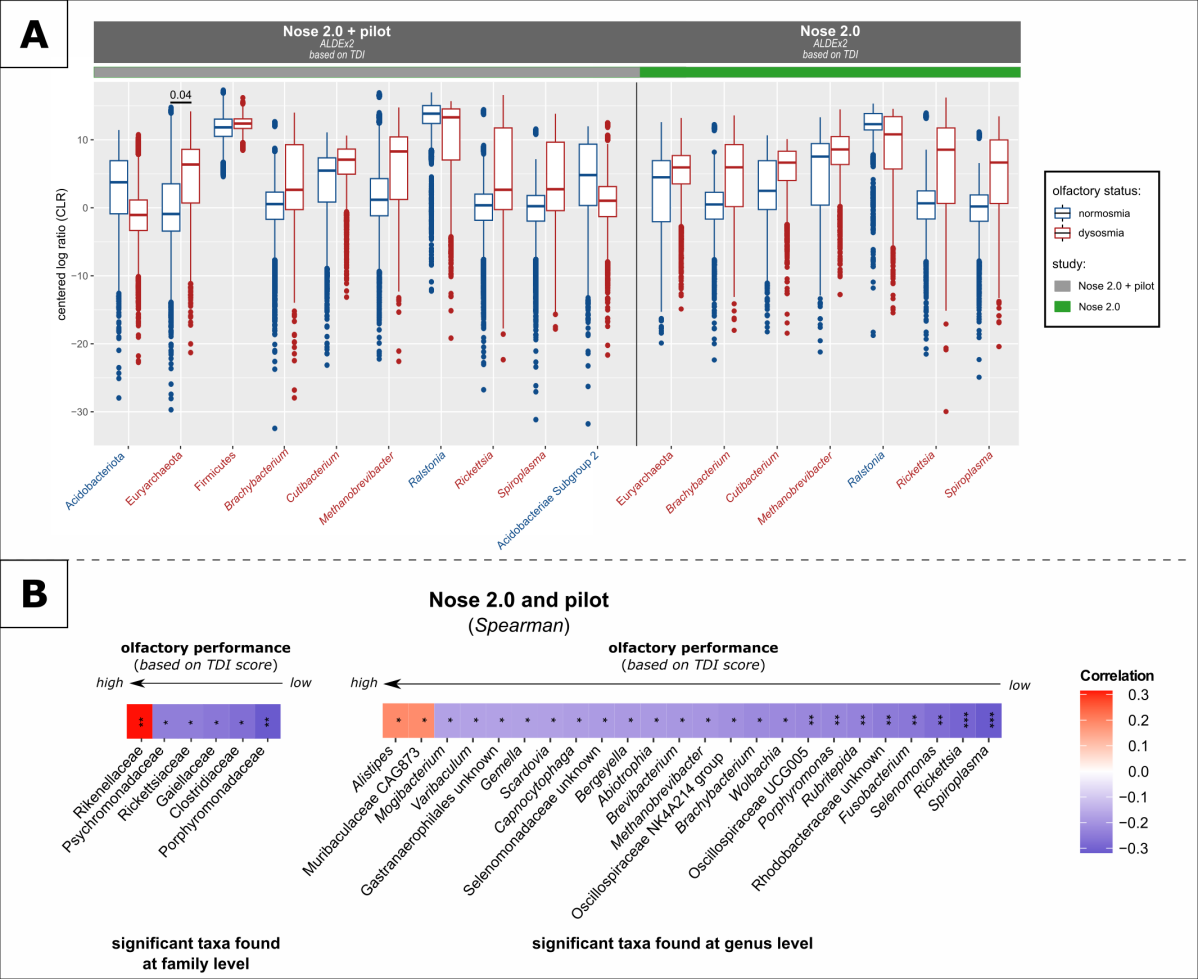
Significantly different taxa found based on olfactory main groups and TDI scores. (**A**) The data were CLR transformed before ALDEx2 was performed. No significant differences were observed in the pilot cohort. For detailed information, see also Table S6. (**B**) Several taxa were positively and negatively correlated with TDI scores (Spearman’s rho correlation; all *P*-values were not adjusted).

The phylum Acidobacteriota (*P* = 0.009, ↑normosmia) and the genus *Ralstonia* (*P* = 0.05, ↑N) were found to be significantly increased in normosmics; thus, these taxa could be associated with overall olfactory function. *Brachybacterium* (*P* = 0.03, ↑dysosmia), *Rickettsia* (*P* = 0.02, ↑D), and *Spiroplasma* (*P* = 0.01, ↑D) were correlated with the dysosmic situation. Notably, the abundance of Euryarchaeota (*q* = 0.04, ↑D; including *Methanobrevibacter*) was strongly associated with dysosmia (Fig. 4).

The results of the analysis based on the underlying single olfactory scores (T, D, and I) further confirm the results based on the major groups with additional taxa found to be associated with normosmics, such as *Corynebacterium* (*P* = 0.003, ↑N), *Delftia* (*P* = 0.003, ↑N), and *Bradyrhizobium* (*P* = 0.01, ↑N) (*P*-values not adjusted; Fig. S5). As already observed for the microbial diversity of the olfactory subgroups, the relative abundance of specific taxa revealed correlations with the TDI score. *Alistipes* [*P* = 0.03, Spearman’s rho (ρ) = 0.206[ and Muribaculaceae CAG873 (*P* = 0.031; ρ = 0.204) were found to be associated with normosmic subjects. In addition to previously mentioned taxa such as *Spiroplasma* (*P* = 0.0002; ρ = −0.346) and *Rickettsia* (*P* = 0.001; ρ = −0.323), oral-associated microbes were also found, including *Fusobacterium* (*P* = 0.003; ρ = −0.282), *Selenomonas* (*P* = 0.001; ρ = −0.302), and *Porphyromonas* (*P* = 0.008; ρ = −0.249) (Fig. 4). Moreover, the predicted community state types did not explain the observed differences in the microbial profiles of the olfactory groups (data not shown).

Similar trends in both cohorts that were found included an increased alpha diversity, indicating more microbial signatures in dysosmic than in normosmic participants with additional fluctuating beta diversity seen in dysosmics. These differences are possibly explained by the presence of biomarkers and especially for dysosmics. Dysosmics were characterized by an increase in the relative abundance of gut- and oral-associated microbes (e.g., *Methanobrevibacter*, *Fusobacterium*, *Porphyromonas*, and *Selenomonas* (33, 37)] and mainly intracellular living microbes [e.g., *Rickettsia* and *Spiroplasma* (67, 68)].

These results raise the question of whether the observed microbial signatures belong to viable and actual inhabitants of the olfactory area or whether these signatures reach this area through respiration—these questions will be addressed later in the manuscript.

### The olfactory area of dysosmics contains many signatures of dead cells

In order to approach the mechanistic question of the microbiome in the olfactory area in normosmics and dysosmics, we concentrated our analyses on the viable [propidium monoazide (PMA)-treated] and active (metatranscriptomics) fractions of the microbial community.

PMA is used to mask freely accessible DNA for subsequent PCR reactions and thus allows one to gain a reliable insight into the intact/viable fraction of microbiomes (31). Many of the abovementioned key findings were still detected after PMA treatment, such as the proportion of archaea (2%) and the most abundant phyla and genera (including the phyla Proteobacteria, Firmicutes, and Actinobacteria and the genera *Ralstonia*, *Corynebacterium*, *Staphylococcus*, and *Corynebacterium*), where most showed similar trends for olfactory groups (Table S7; Data set S2). However, the trends seen for Bacteroidota, Euryarchaeota, *Ralstonia*, and *Dolosigranulum* were exactly the opposite of those observed in the PMA-untreated samples (Fig. S6A).

The analysis of the PMA data set revealed opposite trends for microbial alpha diversity measures as compared with the untreated data set. In untreated samples, as explained above, we observed an increased Shannon index, richness (Chao1), and evenness that correlated with dysosmia; the PMA-treated samples showed decreases in terms of all measured alpha diversity analyses from good to bad olfactory performance. However, the results [Shannon index and richness (Chao1)] were not significant except for microbial evenness (*P* = 0.05; Fig. S6A). In contrast to the previous results on the overall microbial community, the signatures of the viable community did not show significant differences in terms of their beta diversity (Fig. S6A). An analysis of microbial biomarkers for olfactory performance did not reveal significant differences between the olfactory groups using ALDEx2.

Comparing the PMA and non-PMA data set at the genus level, we revealed that 121 unique genera are shared between the data sets (PMA—174 unique genera; non-PMA—316 unique genera). The read counts belonging to normosmia that are shared between both data sets made up 50% and 53%, respectively, whereas the proportion of normosmia and dysosmia in the non-PMA data set was lower in the non-PMA (0.32 or 24% normosmia) than in the PMA (0.85 or 46% normosmia) data set (Fig. S7). Two of the five genera found in the dysosmics, namely, *Rickettsia* and *Spiroplasma*, were found only in non-PMA samples (Fig. S7; Table S8).

All of these results lead us to conclude that dysosmics carry an increased proportion of dead microorganisms as compared with normosmics. To further evaluate the quantity and quality of the dead microbial material, 12 nasal samples (6 normosmia and 6 dysosmia) were selected for further metatranscriptomics analysis.

The information derived from PMA-treated analyses was further confirmed by our metatranscriptomic analysis (Table S9). Again, the alpha diversity showed the same opposite trend as seen in the PMA-treated as compared with untreated samples, and the core microbiome at the phylum level was similar to that detected using the other methods (Fig. S6B).

No feature that was identified as a biomarker for dysosmics in the amplicon data set from the Nose 2.0 study could be found in the metatranscriptomics data set (Fig. 4; Fig. S5; Amplicon, Data set S1-S6). This is likely due to the difficult nature of metatranscriptomics analysis of nasal samples, small sample size, and many unannotated taxa. However, the phylum Actinobacteriota (*P* = 0.045) and the genus *Corynebacterium (P* > 0.05) were found to be indicative for normosmics in the metatranscriptomics output (DeSeq; ALDEx2, data not shown). An analysis of information about the non-bacterial taxonomy (fungi, archaea, and viruses; Data set 8 and 9) and functional capacity did not reveal statistically significant differences between the olfactory groups (Fig. S8; Table S10; Data set 7).

Both the metatranscriptomics and PMA-based 16S rRNA gene analyses revealed a decrease in alpha-diversity measures that correlated with dysosmia. As this result contrasts with the observations made in the classical microbiome analysis (non-PMA-treated samples), we conclude that the olfactory area of dysosmics contains a higher proportion of signatures of dead/non-intact cells as compared with that of normosmics.

### Signatures of dead microbes in the nasal cavity do not come from the gut

An increased relative abundance of butyrate producers in the nasal samples (usually found in the gastrointestinal tract) of dysosmics as compared with normosmics was already indicated by the results of our pilot study (13). These results suggest that dead microbial material is transferred from the gut to the nasal cavity. To determine whether microbes stray from the intestine into the nose, we also investigated the viable fecal microbiome (PMA) of all participants.

**Fig 5:**
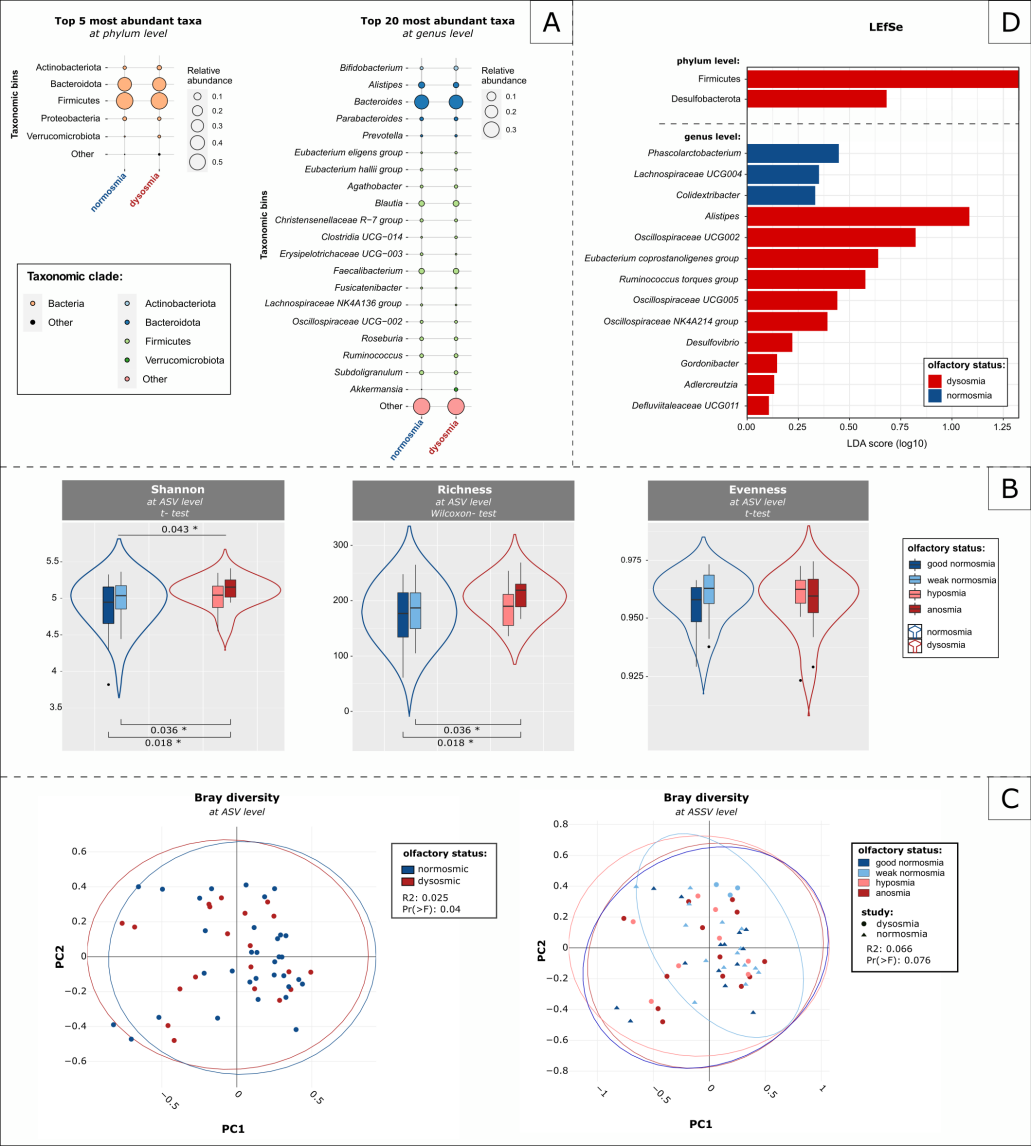
Composition of stool microbiome differs based on olfactory performance. (**A**) Bubble plot showing the most abundant taxa at the phylum and genus levels. (**B**) Alpha diversity indices [Shannon index and richness (Chao1)] were higher in dysosmics as compared with normosmics, except for evenness. (**C**) No clustering was observed based on the olfactory main groups and subgroups. (**D**) The LEfSe analysis revealed that more taxa were associated with dysosmics than with normosmics.

In this microbiome, 99.1% of the observed features belonged to Bacteria and 0.09%, to Archaea, when a universal amplicon sequencing approach was taken. Firmicutes (54.2%), Bacteroidota (36.2%), and Actinobacteriota (3.31%) accounted for around 90% of these features at the phylum level. The genera *Bacteroides* (24.6%), *Alistipes* (4.7%), *Blautia* (4.38%), *Faecalibacterium* (4.1%), *Subdoligranulum* (2.5%), and *Ruminococcus* (2.3%) were defined as the most abundant ones in all samples (Fig. 5; Table S11; Data set S3). In brief, as in the nasal samples (non-PMA), the alpha diversity of the stool samples was significantly increased in dysosmics as compared with in normosmics (Shannon index: *P* = 0.043) with a stepwise decrease in the Shannon index and richness (Chao1) from bad to good olfactory performance (Shannon index/richness (Chao1): AW *P* = 0.036; AG *P* = 0.018; Fig. 5). Although it is not obvious in the PCA plot, the Adonis test yielded a significant result (*P* = 0.04; Fig. 5). Several taxa, including *Alistipes*, *Oscillospiraceae UCG002*, and the *Ruminococcus torques* group, as well as *Colidextribacter* and *Phascolarctobacterium*, were found as putative biomarkers for dysosmia and normosmia, respectively (ALDEx2; full list in Table S12). An analysis of the community state types could not explain the observed differences based on olfactory performance (Fig. S9).

Furthermore, no significant correlations between the key taxa of nasal and stool samples were found (Fig. S10), and source tracking (at ASV and genus levels) did not reveal a substantial overlap in the overall microbial signatures found in gut and nasal samples (0.49% for anosmics, 0.45% for normosmics; genus level).

For completeness, we also analyzed the information on the fungi and archaea from the stool data set (Data set S4 and S5). The fungal communities in both groups consisted mainly of food-borne fungi, including *Saccharomyces* (bread), *Penicillium* (cheese and meat), and *Debaryomyces* (production of vitamin B2 found on food) (Fig. S11). Signatures of *Debaryomyces* (*P* = 0.05) and *Scopulariopsis* (*P* = 0.03; infections including sinusitis) were more common in dysosmics. Almost all archaeal signatures found (32 out of 45 features) belonged to the genus *Methanobrevibacter* (Fig. S11). Due to the nested archaeal PCR approach taken, no differential abundance analysis of the olfactory groups could be performed.

One notable result is that *Methanobrevibacter* signatures were found to be the most abundant archaea in the nasal (PMA and non PMA) and stool samples (universal and archaeal approach) in our study. *Methanobrevibacter* are widely distributed in the human aero-digestive tract. In particular, the archaeal signatures from anosmics clustered with signatures found in fecal samples, indicating a potential overlap (Fig. S12).

### Dysosmics tend to have a more heavily meat-based diet than normosmics

Although one might assume that dietary habits would vary based on the different olfactory performance (i.e., the sense of smell is strongly linked to the sense of taste), we only found tendencies in this regard. The evident trends calculated based on TDI scores (*P* < 0.05; Spearman’s rho) included mainly the food categories meat (*P* = 0.002; ρ = −0.431) and fish (*P* = 0.018; ρ = −0.339), which were eaten more frequently in dysosmics, whereas the intake of fruits (*P* = 0.012; ρ = 0.359) and legumes (*P* = 0.012; ρ = 0.359) was higher in normosmics. When considering the microbiome aspect, only the processed meat intake (dysosmics; *P* = 0.025; ρ = −0.315) was significantly correlated with the fungal community of the dysosmics’ stool samples ( Fig. 13A, B) and the meat consumption, with the nasal microbiome of dysosmics (*P* = 0.018; non PMA, Fig. 13C, D). Several trends were also observed for nutrients (e.g., fatty acids, vitamins, and carbohydrates), but only the essential omega-3 fatty acid ⍺-linolenic acid (*q* = 0.03; ρ = 0.495) had a significantly higher correlation with olfactory function than dysfunction after *P*-value correction (Tables S14). Interestingly, omega-3 fatty acids, in general, have been associated with an improvement in olfactory function in other studies (69, 70). All in all, normosmic participants seem to eat “healthier” (more fruits, less meat) than the dysosmics.

In addition to the amplicon information, the metabolites in the stool samples were determined. The presence of the metabolites *D*-fructose (*P* = 0.037; ρ = 0.299), lactulose (*P* = 0.002; ρ = 0.425), hypoxanthine (*P* = 0.037; ρ = 0.299), and nicotinic acid (*P* = 0.007; ρ = 0.379) were associated with better olfactory performance. Furthermore, an analysis of correlations between the metabolites and the key taxa found in stool and nasal samples revealed trends (*P* < 0.05) for both normosmics and dysosmics ( Table S1 to S15; Fig. S14).

Even though we detected trends, the changes in the diet could not explain the differences observed in the microbiome composition. However, in general, it seems as though normosmics are more likely to follow a vegetarian diet.

## DISCUSSION

The full spectrum of the physiological effects of olfactory loss is still largely unknown, and, according to our findings, the olfactory microbiome is also affected or even involved in this devastating condition. By carrying out two independent studies, we could show that the microbiome composition varies with olfactory capacity. This was found to be mainly associated with an increased diversity in the dead/non-functional microbial cells at the olfactory epithelium. The source of these dead/non-functioning microbial cells has not been fully elucidated, but we found evidence that the oral cavity, rather than the gut, is a possible source.

The overall composition of the microbial community in the olfactory area varied as a function of olfactory performance. The same trends were observed in this study (Nose 2.0) and the pilot study, indicating reproducible results. Although the olfactory groups overlapped in terms of their microbial composition, the composition in dysosmics was more scattered with respect to beta diversity, suggesting that the microbial composition was not as stable as in the normosmics. Consistently, the analysis of the alpha diversity also revealed higher microbial richness (Chao1) (more different taxa) in samples with reduced olfactory performance.

In contrast to our results, Biswas et al. (2020) reported a loss of diversity in individuals with poor olfactory function; however, in this case, the authors investigated the microbiome of the sinuses, and thus, the findings are not fully comparable (23). Other diseases of the upper respiratory tract have also been reported to be associated with lower levels of microbial diversity [e.g., chronic Rhinosinusitis (71) and cystic fibrosis (72)]. Only allergic rhinitis has displayed the same trend as we found in our study, with higher levels of microbial diversity observed in diseased as compared with healthy subjects (73).

Interestingly, analyses of the data on viable/intact cells in the nasal samples revealed a nearly opposite trend, suggesting that dysosmics carry a higher load of dead/non-functional microorganisms. This result was also obtained when taking two independent molecular approaches, namely, PMA-treatment and metatranscriptomics. This finding is further supported by the fact that several taxa were found in both PMA-treated and untreated nasal samples, respectively; overlaps were observed among the groups at a genus level but also among an enormous number of the signatures that were specific for PMA-untreated samples (62% of total non-PMA signatures). Of the genera found in the non-PMA-specific data set, only 24% belonged to the normosmic samples, while the normosmic proportion of the 121 genera found in both data sets (PMA and non-PMA) accounted for about 50%.

The excess number of ASVs might have been introduced from other body parts or the external environment via inhalation. However, the impact of the gut microbiome was clarified by source tracking, and the results indicate that the dead material originated from other sources, such as other nasal areas, skin, the oral cavity, or the environment. We could even find evidence that in particular, oral (or specifically subgingival) taxa such as *Gemella*, *Capnocytophaga*, *Fusobacterium*, and *Porphyromonas* were enriched in the olfactory area of dysosmics. Some of these have even been associated with periodontitis (e.g., *Fusobacterium* or *Porphyromona*s) in other studies (74).

Chemosensory conditions have been associated with oral conditions in earlier studies (75) and, in particular, with halitosis (e.g., *Fusobacterium* and *Porphyromonas*) that was associated with smell and taste disturbance (76). Thus, our findings might indicate problematic dental conditions that would require additional and more thorough analyses. In any case, the higher load of dead/non-functional material might be due to a potentially reduced mucociliary clearance (i.e., where the dead material can no longer be transported toward the throat and/or microbes can migrate into the nasal cavity from lower body parts) in dysosmics.

Notably, dysosmics displayed a higher number of signatures for (dead/non-functional) intracellular microbes (e.g., *Spiroplasma* and *Rickettsia* (67, 68)); the reason for this observation is unclear, and the higher number of signatures is not correlated with increased inflammation. Although the cause of dysosmia in our participants was an infection, no significant difference in inflammatory markers (glucose, CRP, and IL6-L1) between the olfactory groups was observed. This result suggests that the initial inflammation/disease had been overcome, and the long-term smell loss was not driven by an active inflammation.

Interestingly, we found a significant difference in the stool microbiome based on olfactory performance. This stepwise decrease in alpha diversity from low to high olfactory performance and the distinctive microbial composition (normosmia: *Bacteroides*, *Colidextribacter*, and *Phascolarctobacterium*; dysosmia: *Alistipes*, Oscillospiraceae UCG 002, and *Ruminococcus torques* group) might be explained by changes in the diet, favoring different kinds of microbes. Overall, the dysosmics’ diet was characterized by a rather higher consumption of (processed) meat and fish as compared with the “healthier” diet of normosmics (i.e., more fruits and legumes). Furthermore, we found a decreased intake of the omega-3 fatty acid, alpha-linolenic acid, in dysosmics. Consistent with our findings, other studies could show an improvement in olfactory function as a result of omega-3 fatty acid supplementation (69, 70). Since omega-3 fatty acids have neuroprotective and antioxidant effects, as well as boost the anti-inflammatory amino acid production, supplementation might serve as a putative therapy option for olfactory loss (77, 78).

### Study limitations

To draw appropriate conclusions about the nasal microbiome and olfactory performance in humans, the olfactory mucosa was the sampling site of choice. This is a tiny area (8–10 mm) on the ceiling of the nasal cavity. Hence, the small volume of the specimens obtained from this area presented a major challenge, which was tackled for the first time in this study. Major issues that needed to be overcome were to find a less-contamination-prone, swab-less sampling procedure and a highly efficient DNA extraction method, both of which needed to be tested and established. However, the small amount of biomass retrieved still presented problems during the subsequent analyses. Thus, only 12 samples (6 from 33 normosmics plus 6 from 20 dysosmics) were suitable for RNA sequencing and did not offer the possibility to re-run and improve the analyses. Therefore, we cannot make any statements about the link between olfaction and the functional profile of the microbiome; we were restricted to drawing conclusions from the taxonomic information.

We are aware that the overall number of participants (in total *n* = 53) is still low; the recruiting process was difficult due to a discrepancy in the personal olfactory perception of the study participants and the outcome of the sniffing-stick-based olfactory measurements. However, we were still able to include more participants with dysosmia than in our pilot study, which was included in order to run comparative analyses and to increase the total number of individuals analyzed. To minimize the study bias, the pilot cohort was completely re-evaluated along with the new cohort.

The study design did not include analyses of the aerobiome of the environment of the study participants. Therefore, no conclusions can be made about the aerobiome as the source of the dead microbial fraction.

### Ongoing work

The results of our study show that the nasal microbiome differs according to olfactory performance and that the load of dead cells is significantly higher in dysosmics. Several independent studies have shown that the sense of smell can be improved by performing olfactory training (79–81), but potential effects on the nasal microbiome have never been investigated. In the future, we plan to investigate whether the smell training can be used to revert the “dysosmic” microbiome to a more favorable “normosmic” one (e.g., less dead material) in a longitudinal study using three time points [before (TP0), during (TP1), and after (TP3) smell training]. Furthermore, we want to use the gained knowledge to combine two distinct fields of the human life sciences, namely, microbiome research and neuroimaging, to investigate whether a reorganization of the brain structure takes place and to increase predictive and therapeutic opportunities. In addition, future studies should also consider exposure to the aerobiome via inhalation as a potential source of the dead microbial fraction of the olfactory microbiome.

### Conclusion

People suffering from a loss of smell display a higher burden of dead microbial material near their olfactory receptor cells. The upper respiratory tract is equipped with highly efficient transport mechanisms, the so-called mucociliary clearance defense mechanism [reviewed in reference (15)], which is responsible for the efficient removal of various kinds of material. This clearance process appears to be impaired in dysosmic subjects, which could lead to the increased migration and accumulation of microbes from other body sites or the external environment into the nasal passages.

We were able to exclude the gut as the source of these signatures but linked specific periodontitis-associated microbial signatures to dysosmia. Investigating the relationship between olfactory function, the oral microbiome, and the functionality of mucociliary clearance may help researchers understand the reason for the high burden of non-functioning microbes in the olfactory mucosa of dysosmics.

## Data Availability

Raw data obtained by amplicon sequencing (universal, archaeal, and fungal approaches) as well as metatranscriptomics can be found in the ENA Archive: Project Number PRJEB57226. Supplemental data sets (after preprocessing: decontam, removal of controls, mitochondrial and unassigned features, features with less than two reads, and SRS normalization) are given in the supplemental material. The commands and settings used for pre-processing the amplicon data are given in the supplemental text.

## SUPPLEMENTAL MATERIAL

Supplemental figures spectrum.01549-23-s0001.pdf Figures S1 to S14.

Supplemental text spectrum.01549-23-s0002.pdf Commands and settings for data pre-processing (amplicon).

Supplemental data sets spectrum.01549-23-s0003.xls Data sets S1 to S9.

Supplemental tables spectrum.01549-23-s0004.xlsx Tables S1 to S15.

ASM does not own the copyrights to Supplemental Material that may be linked to, or accessed through, an article. The authors have granted ASM a non-exclusive, world-wide license to publish the Supplemental Material files. Please contact the corresponding author directly for reuse.

## References

[1] Croy I, Nordin S, Hummel T. 2014. Olfactory disorders and quality of life--an updated review. Chem Senses 39:185–194. doi:10.1093/chemse/bjt072 24429163

[2] Small DM, Prescott J. 2005. Odor/taste integration and the perception of flavor. Exp Brain Res 166:345–357. doi:10.1007/s00221-005-2376-9 16028032

[3] Pekala K, Chandra RK, Turner JH. 2016. Efficacy of olfactory training in patients with olfactory loss: a systematic review and meta-analysis. Int Forum Allergy Rhinol 6:299–307. doi:10.1002/alr.21669 26624966 PMC4783272

[4] Boesveldt S, Postma EM, Boak D, Welge-Luessen A, Schöpf V, Mainland JD, Martens J, Ngai J, Duffy VB. 2017. Anosmia-a clinical review. Chem Senses 42:513–523. doi:10.1093/chemse/bjx025 28531300 PMC5863566

[5] Beecher K, St John JA, Chehrehasa F. 2018. Factors that modulate olfactory dysfunction. Neural Regen Res 13:1151–1155. doi:10.4103/1673-5374.235018 30028314 PMC6065237

[6] Mellert TK, Getchell ML, Sparks L, Getchell TV. 1992. Characterization of the immune barrier in human olfactory mucosa. Otolaryngol Head Neck Surg 106:181–188. 1738551

[7] Kimmelman CP. 1993. Clinical review of olfaction. Am J Otolaryngol 14:227–239. doi:10.1016/0196-0709(93)90065-f 8214314

[8] Sahin-Yilmaz A, Naclerio RM. 2011. Anatomy and physiology of the upper airway. Proc Am Thorac Soc 8:31–39. doi:10.1513/pats.201007-050RN 21364219

[9] Pluznick JL, Protzko RJ, Gevorgyan H, Peterlin Z, Sipos A, Han J, Brunet I, Wan LX, Rey F, Wang T, Firestein SJ, Yanagisawa M, Gordon JI, Eichmann A, Peti-Peterdi J, Caplan MJ. 2013. Olfactory receptor responding to gut microbiota-derived signals plays a role in renin secretion and blood pressure regulation. Proc Natl Acad Sci U S A 110:4410–4415. doi:10.1073/pnas.1215927110 23401498 PMC3600440

[10] Liberles SD, Horowitz LF, Kuang D, Contos JJ, Wilson KL, Siltberg-Liberles J, Liberles DA, Buck LB. 2009. Formyl peptide receptors are candidate chemosensory receptors in the vomeronasal organ. Proc Natl Acad Sci U S A 106:9842–9847. doi:10.1073/pnas.0904464106 19497865 PMC2690606

[11] Tizard I, Skow L. 2021. The olfactory system: the remote-sensing arm of the immune system. Anim Health Res Rev 22:14–25. doi:10.1017/S1466252320000262 33926605

[12] Liang F. 2020. Sustentacular cell enwrapment of olfactory receptor neuronal dendrites: an update. Genes (Basel) 11:493. doi:10.3390/genes11050493 32365880 PMC7291085

[13] Koskinen K, Reichert JL, Hoier S, Schachenreiter J, Duller S, Moissl-Eichinger C, Schöpf V. 2018. The nasal microbiome mirrors and potentially shapes olfactory function. Sci Rep 8:1296. doi:10.1038/s41598-018-19438-3 29358754 PMC5778015

[14] Koskinen K, Pausan MR, Perras AK, Beck M, Bang C, Mora M, Schilhabel A, Schmitz R, Moissl-Eichinger C. 2017. First insights into the diverse human archaeome: specific detection of archaea in the gastrointestinal tract, lung, and nose and on skin. mBio 8:e00824-17. doi:10.1128/mBio.00824-17 29138298 PMC5686531

[15] Kumpitsch C, Koskinen K, Schöpf V, Moissl-Eichinger C. 2019. The microbiome of the upper respiratory tract in health and disease. BMC Biol 17:87. doi:10.1186/s12915-019-0703-z 31699101 PMC6836414

[16] Mahdavinia M, Keshavarzian A, Tobin MC, Landay AL, Schleimer RP. 2016. A comprehensive review of the nasal microbiome in chronic rhinosinusitis (CRS). Clin Exp Allergy 46:21–41. doi:10.1111/cea.12666 26510171 PMC4715613

[17] Pellegrino R, Mainland JD, Kelly CE, Parker JK, Hummel T. 2021. Prevalence and correlates of parosmia and phantosmia among smell disorders. Chem Senses 46:bjab046. doi:10.1093/chemse/bjab046 34698820 PMC8633731

[18] Elmassry MM, Piechulla B. 2020. Volatilomes of bacterial infections in humans. Front Neurosci 14:257. doi:10.3389/fnins.2020.00257 32269511 PMC7111428

[19] Creative Commons Attribution 3.0 Unported license. n.d. Servier medical art. Nasal cavity. Available from: https://smart.servier.com/smart_image/nasal-cavity/

[20] Creat Commons Attrib 30 Unported Licens. n.d. Servier medical art. Olfactory bulb. Available from: https://smart.servier.com/smart_image/olfactory-bulb/

[21] N.d. Freepik by Brgfx. Microbes. Available from: https://www.freepik.com/free-vector/different-types-bacteria-intestines_2480485.htm#query=bacteria&position=11&from_view=search&track=sph

[22] Inkscape. 2020. Inkscape project. Available from: https://inkscape.org

[23] Biswas K, Wagner Mackenzie B, Ballauf C, Draf J, Douglas RG, Hummel T. 2020. Loss of bacterial diversity in the sinuses is associated with lower smell discrimination scores. Sci Rep 10:16422. doi:10.1038/s41598-020-73396-3 33009469 PMC7532173

[24] Lighthart B. 2000. Mini-review of the concentration variations found in the alfresco atmospheric bacterial populations. Aerobiologia (Bologna) 16:7–16. doi:10.1023/A:1007694618888

[25] Dickson RP, Erb-Downward JR, Martinez FJ, Huffnagle GB. 2016. The microbiome and the respiratory tract. Annu Rev Physiol 78:481–504. doi:10.1146/annurev-physiol-021115-105238 26527186 PMC4751994

[26] Wanner A, Salathé M, O’Riordan TG. 1996. Mucociliary clearance in the airways. Am J Respir Crit Care Med 154:1868–1902. doi:10.1164/ajrccm.154.6.8970383 8970383

[27] Hummel T, Sekinger B, Wolf SR, Pauli E, Kobal G. 1997. “Sniffin” Sticks’: Olfactory performance assessed by the combined testing of odor identification odor discrimination and olfactory threshold. In Chemical senses. Vol. 22. 10.1093/chemse/22.1.39 9056084

[28] Oleszkiewicz A, Schriever VA, Croy I, Hähner A, Hummel T. 2019. Updated sniffin’ sticks normative data based on an extended sample of 9139. Eur Arch Otorhinolaryngol 276:719–728. doi:10.1007/s00405-018-5248-1 30554358 PMC6411676

[29] Haftenberger M, Heuer T, Heidemann C, Kube F, Krems C, Mensink GBM. 2010. Relative validation of a food frequency questionnaire for national health and nutrition monitoring. Nutr J 9:36. doi:10.1186/1475-2891-9-36 20840739 PMC2945984

[30] Denkwerkzeuge D. 2020. Software:nut.s science V1.32.79. Vienna. www.nutritional-software.at.

[31] Nocker A, Sossa-Fernandez P, Burr MD, Camper AK. 2007. Use of propidium monoazide for live/dead distinction in microbial ecology. Appl Environ Microbiol 73:5111–5117. doi:10.1128/AEM.02987-06 17586667 PMC1951001

[32] Pausan MR, Csorba C, Singer G, Till H, Schöpf V, Santigli E, Klug B, Högenauer C, Blohs M, Moissl-Eichinger C. 2019. Exploring the archaeome: detection of archaeal signatures in the human body. Front Microbiol 10:2796. doi:10.3389/fmicb.2019.02796 31866971 PMC6906140

[33] Kumpitsch C, Moissl-Eichinger C, Pock J, Thurnher D, Wolf A. 2020. Preliminary insights into the impact of primary radiochemotherapy on the salivary microbiome in head and neck squamous cell carcinoma. Sci Rep 10:16582. doi:10.1038/s41598-020-73515-0 33024215 PMC7538973

[34] Klymiuk I, Bambach I, Patra V, Trajanoski S, Wolf P. 2016. 16S based microbiome analysis from healthy subjects' skin swabs stored for different storage periods reveal phylum to genus level changes. Front Microbiol 7:2012. doi:10.3389/fmicb.2016.02012 28066342 PMC5167739

[35] Callahan BJ, McMurdie PJ, Rosen MJ, Han AW, Johnson AJA, Holmes SP. 2016. DADA2: high-resolution sample inference from illumina amplicon data. Nat Methods 13:581–583. doi:10.1038/nmeth.3869 27214047 PMC4927377

[36] Robeson MS, O’Rourke DR, Kaehler BD, Ziemski M, Dillon MR, Foster JT, Bokulich NA. 2021. RESCRIPT: reproducible sequence taxonomy reference database management. PLoS Comput Biol 17:e1009581. doi:10.1371/journal.pcbi.1009581 34748542 PMC8601625

[37] Kumpitsch C, Fischmeister FPS, Mahnert A, Lackner S, Wilding M, Sturm C, Springer A, Madl T, Holasek S, Högenauer C, Berg IA, Schoepf V, Moissl-Eichinger C. 2021. Reduced B12 uptake and increased gastrointestinal formate are associated with archaeome-mediated breath methane emission in humans. Microbiome 9:193. doi:10.1186/s40168-021-01130-w 34560884 PMC8464155

[38] Quast C, Pruesse E, Yilmaz P, Gerken J, Schweer T, Yarza P, Peplies J, Glöckner FO. 2013. The SILVA ribosomal RNA gene database project: improved data processing and web-based tools. Nucleic Acids Res 41:D590–6. doi:10.1093/nar/gks1219 23193283 PMC3531112

[39] Bokulich NA, Kaehler BD, Rideout JR, Dillon M, Bolyen E, Knight R, Huttley GA, Gregory Caporaso J. 2018. Optimizing taxonomic classification of marker-gene amplicon sequences with QIIME 2’s Q2-feature-classifier plugin. Microbiome 6:90. doi:10.1186/s40168-018-0470-z 29773078 PMC5956843

[40] Davis NM, Proctor DM, Holmes SP, Relman DA, Callahan BJ. 2018. Simple statistical identification and removal of contaminant sequences in marker-gene and metagenomics data. Microbiome 6:226. doi:10.1186/s40168-018-0605-2 30558668 PMC6298009

[41] Heidrich V, Karlovsky P, Beule L. 2021. SRS’ R package and ‘Q2-Srs’ QIIME 2 Plugin: Normalization of Microbiome data using Scaling with ranked Subsampling (SRS). Appl Sci 11:11473. doi:10.3390/app112311473

[42] Paulson J, Reeder J, Huang M. 2022. microbiomeExplorer: Microbiome exploration App. doi:10.18129/B9.bioc.microbiomeExplorer

[43] Team Rs. 2020. Rstudio: integrated development environment for R. RStudio, PBC, Boston, MA. Available from: http://www.rstudio.com/

[44] Mauri M, Elli T, Caviglia G, Uboldi G, Azzi M. 2017. RAWGraphs: a visualisation platform to create open outputs, p 1–28. In Proceedings of the 12th Biannual Conference on Italian SIGCHI chapter. Vol. 28. New York, NY: ACM.

[45] Asya K. 2021. Lefser: R implementation of the LEFSE method for microbiome biomarker discovery. Rpackage version 140

[46] Fernandes AD, Reid JN, Macklaim JM, McMurrough TA, Edgell DR, Gloor GB. 2014. Unifying the analysis of high-throughput sequencing datasets: characterizing RNA-Seq, 16S rRNA gene sequencing and selective growth experiments by compositional data analysis. Microbiome 2:15. doi:10.1186/2049-2618-2-15 24910773 PMC4030730

[47] Gloor GB, Macklaim JM, Fernandes AD. 2016. Displaying variation in large datasets: a visual summary of effect sizes. J Comp Graph Stats 25:971–979. doi:10.1080/10618600.2015.1131161

[48] Andrews S. 2010. FastQC a quality control tool for high throughput sequence data. Available from: http://www.bioinformatics.babraham.ac.uk/projects/fastqc

[49] Bolger AM, Lohse M, Usadel B. 2014. Trimmomatic: a flexible trimmer for illumina sequence data. Bioinform 30:2114–2120. doi:10.1093/bioinformatics/btu170 PMC4103590 24695404

[50] Langmead B, Salzberg SL. 2012. Fast gapped-read alignment with bowtie 2. Nat Methods 9:357–359. doi:10.1038/nmeth.1923 22388286 PMC3322381

[51] Huson DH, Auch AF, Qi J, Schuster SC. 2007. MEGAN analysis of metagenomic data. Genome Res 17:377–386. doi:10.1101/gr.5969107 17255551 PMC1800929

[52] Li D, Liu CM, Luo R, Sadakane K, Lam TW. 2015. MEGAHIT: an ultra-fast single-node solution for large and complex metagenomics assembly via succinct de bruijn graph. Bioinformatics 31:1674–1676. doi:10.1093/bioinformatics/btv033 25609793

[53] Wu YW, Tang YH, Tringe SG, Simmons BA, Singer SW. 2014. MaxBin: an automated binning method to recover individual genomes from metagenomes using an expectation-maximization algorithm. Microbiome 2:26. doi:10.1186/2049-2618-2-26 25136443 PMC4129434

[54] Olm MR, Brown CT, Brooks B, Banfield JF. 2017. DRep: a tool for fast and accurate genomic comparisons that enables improved genome recovery from metagenomes through de-replication. ISME J 11:2864–2868. doi:10.1038/ismej.2017.126 28742071 PMC5702732

[55] Kreuzer K, Reiter A, Birkl-Töglhofer AM, Dalkner N, Mörkl S, Mairinger M, Fleischmann E, Fellendorf F, Platzer M, Lenger M, et al.. 2022. The PROVIT study-effects of multispecies probiotic add-on treatment on metabolomics in major depressive disorder-A randomized, placebo-controlled trial. Metabolites 12:770. doi:10.3390/metabo12080770 36005642 PMC9414726

[56] Zhou Q, Kerbl-Knapp J, Zhang F, Korbelius M, Kuentzel KB, Vujić N, Akhmetshina A, Hörl G, Paar M, Steyrer E, Kratky D, Madl T. 2021. Metabolomic profiles of mouse tissues reveal an interplay between aging and energy metabolism. Metabolites 12:17. doi:10.3390/metabo12010017 35050139 PMC8779655

[57] Oksanen J, Blanchet FG, Kindt R, Legendre P, Minchin PR, O’Hara R, et al.. 2022. Vegan: community ecology package. https://cran.r-project.org/package=vegan.

[58] Corp IBM. 2019. IBM SPSS statistics for windows. IBM Corp, Armonk, NY.

[59] ggpubr KA. 2020. “ggplot2” based publication ready plots. Available from: https://cran.r-project.org/package=ggpubr

[60] Wickham H, Averick M, Bryan J, Chang W, McGowan L, François R, Grolemund G, Hayes A, Henry L, Hester J, Kuhn M, Pedersen T, Miller E, Bache S, Müller K, Ooms J, Robinson D, Seidel D, Spinu V, Takahashi K, Vaughan D, Wilke C, Woo K, Yutani H. 2019. Welcome to the tidyverse. JOSS 4:1686. doi:10.21105/joss.01686

[61] Rinker TW. 2021. Textshape: tools for reshaping text. Buffalo, New York. Available from: https://github.com/trinker/textshape

[62] Wickham H, François R, Henry L, Müller K. 2022. dplyr: a grammar of data manipulation. Available from: https://cran.r-project.org/package=dplyr

[63] Müller K, Wickham H. 2021. tibble: simple data frames. Available from: https://cran.r-project.org/package=tibble

[64] Wickham H. 2016. ggplot2: elegant graphics for data analysis. Springer-Verlag, New York. Available from: https://ggplot2.tidyverse.org

[65] Knights D, Kuczynski J, Charlson ES, Zaneveld J, Mozer MC, Collman RG, Bushman FD, Knight R, Kelley ST. 2011. Bayesian community-wide culture-independent microbial source tracking. Nat Methods 8:761–763. doi:10.1038/nmeth.1650 21765408 PMC3791591

[66] Letunic I, Bork P. 2021. Interactive tree of life (iTOL) V5: an online tool for phylogenetic tree display and annotation. Nucleic Acids Res 49:W293–W296. doi:10.1093/nar/gkab301 33885785 PMC8265157

[67] Cisak E, Wójcik-Fatla A, Zając V, Sawczyn A, Sroka J, Dutkiewicz J. 2015. Spiroplasma - an emerging arthropod-borne pathogen? Ann Agric Environ Med 22:589–593. doi:10.5604/12321966.1185758 26706960

[68] Helminiak L, Mishra S, Kim HK. 2022. Pathogenicity and virulence of rickettsia. Virulence 13:1752–1771. 36208040 10.1080/21505594.2022.2132047 PMC9553169

[69] Yan CH, Rathor A, Krook K, Ma Y, Rotella MR, Dodd RL, Hwang PH, Nayak JV, Oyesiku NM, DelGaudio JM, Levy JM, Wise J, Wise SK, Patel ZM. 2020. Effect of Omega-3 supplementation in patients with smell dysfunction following endoscopic Sellar and Parasellar tumor resection: a multicenter prospective randomized controlled trial. Neurosurgery 87:E91–E98. doi:10.1093/neuros/nyz559 31950156 PMC7360874

[70] Gopinath B, Sue CM, Flood VM, Burlutsky G, Mitchell P. 2015. Dietary intakes of fats, fish and nuts and olfactory impairment in older adults. Br J Nutr 114:240–247. doi:10.1017/S0007114515001257 26079067

[71] De Boeck I, Wittouck S, Martens K, Claes J, Jorissen M, Steelant B, van den Broek MFL, Seys SF, Hellings PW, Vanderveken OM, Lebeer S. 2019. Anterior nares diversity and pathobionts represent sinus microbiome in chronic rhinosinusitis. mSphere 4:e00532-19. doi:10.1128/mSphere.00532-19 31776238 PMC6881717

[72] Cuthbertson L, Walker AW, Oliver AE, Rogers GB, Rivett DW, Hampton TH, Ashare A, Elborn JS, De Soyza A, Carroll MP, Hoffman LR, Lanyon C, Moskowitz SM, O’Toole GA, Parkhill J, Planet PJ, Teneback CC, Tunney MM, Zuckerman JB, Bruce KD, van der Gast CJ. 2020. Lung function and microbiota diversity in cystic fibrosis. Microbiome 8:45. doi:10.1186/s40168-020-00810-3 32238195 PMC7114784

[73] Choi C, Poroyko V, Watanabe S, Jiang D, Lane J, deTineo M, Baroody FM, Naclerio RM, Pinto JM. 2014. Seasonal allergic rhinitis affects sinonasal microbiota. Am J Rhinol Allergy 28:281–286. doi:10.2500/ajra.2014.28.4050 25197913 PMC4101129

[74] How KY, Song KP, Chan KG. 2016. Porphyromonas gingivalis: an overview of periodontopathic pathogen below the gum line. Front Microbiol 7:53. doi:10.3389/fmicb.2016.00053 26903954 PMC4746253

[75] Batisse C, Bonnet G, Eschevins C, Hennequin M, Nicolas E. 2017. The influence of oral health on patients' food perception: a systematic review. J Oral Rehabil 44:996–1003. doi:10.1111/joor.12535 28600840

[76] Schertel Cassiano L, Leite FRM, López R, Fjaeldstad AW, Nascimento GG. 2023. The association between halitosis and chemosensory disorders: a systematic review. Oral Dis 29:369–375. doi:10.1111/odi.13823 33660384

[77] Gladman SJ, Huang W, Lim SN, Dyall SC, Boddy S, Kang JX, Knight MM, Priestley JV, Michael-Titus AT. 2012. Improved outcome after peripheral nerve injury in mice with increased levels of endogenous ω-3 polyunsaturated fatty acids. J Neurosci 32:563–571. doi:10.1523/JNEUROSCI.3371-11.2012 22238091 PMC6621061

[78] Figueroa JD, De Leon M. 2014. Neurorestorative targets of dietary long-chain omega-3 fatty acids in neurological injury. Mol Neurobiol 50:197–213. doi:10.1007/s12035-014-8701-1 24740740 PMC4183712

[79] Altundag A, Cayonu M, Kayabasoglu G, Salihoglu M, Tekeli H, Saglam O, Hummel T. 2015. Modified olfactory training in patients with postinfectious olfactory loss. Laryngoscope 125:1763–1766. doi:10.1002/lary.25245 26031472

[80] Damm M, Pikart LK, Reimann H, Burkert S, Göktas Ö, Haxel B, Frey S, Charalampakis I, Beule A, Renner B, Hummel T, Hüttenbrink K-B. 2014. Olfactory training is helpful in postinfectious olfactory loss: a randomized, controlled, multicenter study. Laryngoscope 124:826–831. doi:10.1002/lary.24340 23929687

[81] Konstantinidis I, Tsakiropoulou E, Constantinidis J. 2016. Long term effects of olfactory training in patients with post-infectious olfactory loss. Rhinol J 54:170–175. doi:10.4193/Rhino15.264 27017331

