# Supplementary material for: Reduced olfactory performance is associated with changed microbial diversity, oralization, and accumulation of dead biomaterial in the nasal olfactory area
Source: Microbiol Spectr. 2024 Jan 9;12(2):e01549-23. doi: 10.1128/spectrum.01549-23 (PMC10846256; doi:10.1128/spectrum.01549-23)
Supplement: Supplemental figures — Figures S1 to S14. [file spectrum.01549-23-s0001.pdf]

Supplementary Figures 1-14

Reduced olfactory performance is associated with changed microbial diversity, oralization and accumulation of dead biomaterial in the nasal olfactory area

Christina Kumpitsch[1], Florian Ph. S. Fischmeister[2,3,6], Sonja Lackner[4], Sandra Holasek[4], Tobias Madl[5,6], Hansjörg Habisch[5], Axel Wolf[7], Veronika Schöpf[3], Christine Moissl-Eichinger[1,6]

[1] Diagnostic and Research Institute of Hygiene, Microbiology and Environmental Medicine, Medical University of Graz, Graz 8010, Austria

[2] Department of Psychology, University of Graz, Graz 8010, Austria

[3] Department of Biomedical Imaging and Image-guided Therapy, Medical University of Vienna, Vienna, Austria

[4] Otto Loewi Research Center, Division of Immunology, Medical University of Graz, Graz 8010, Austria

[5] Gottfried Schatz Research Center for Cell Signaling, Metabolism and Ageing, Molecular Biology and Biochemistry, Research Unit Integrative Structural Biology, Medical University of Graz, 8010 Graz, Austria

[6] BioTechMed, Graz, Graz 8010, Austria

[7] Department of Otorhinolaryngology, Medical University of Graz, Graz, Austria

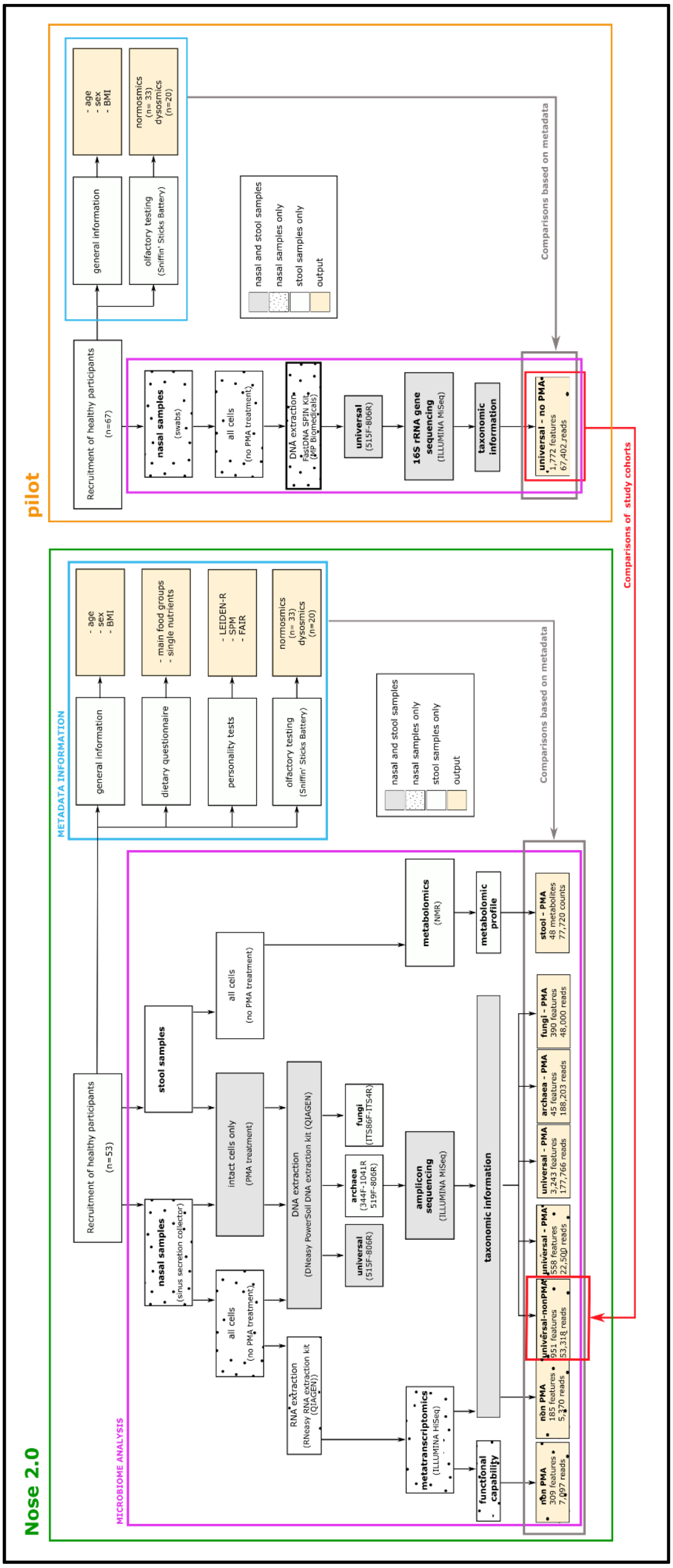

Supplementary Figure 1. Schematic overview of methodology used in the studies. Nasal (and stool) samples were collected; amplicon sequencing, metatranscriptomics and metabolomics were performed; and results were compared based on the metadata and study cohort. The boxes were color-coded as follows: dotted - nasal samples only, clear - stool samples only, grey - nasal and stool samples, orange – outputs.

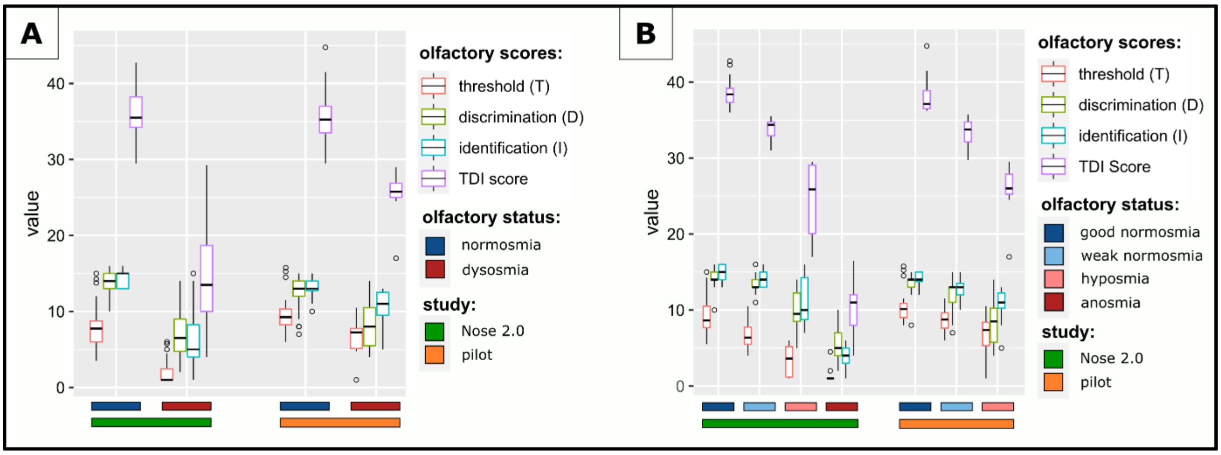

Supplementary Figure 2. Olfactory performance assessed via the standardized TDI score. **A)** The TDI scores of all participants based on the main olfactory groups (normosmia: TDI ≥ 31). **B)** For a more detailed analysis, normosmia as well as dysosmia were further divided into subcategories: good normosmia (TDI ≥ 41.25), weak normosmia (41.25 < TDI > 30.5), hyposmia (30.5 ≤ TDI > 16), and anosmia (TDI ≤ 16).

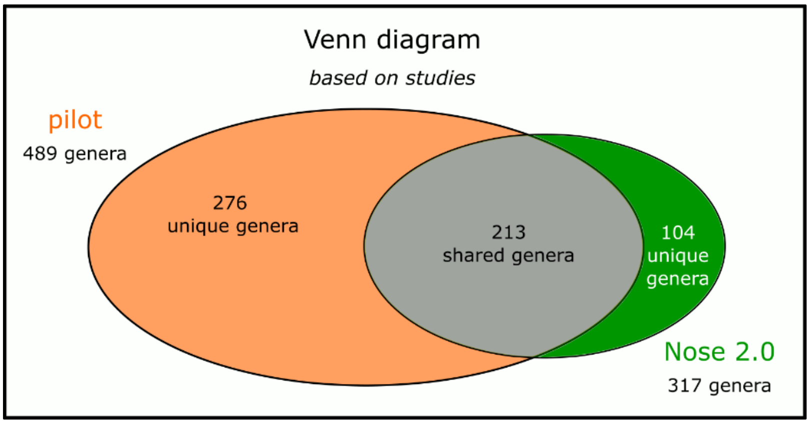

Supplementary Figure 3. Venn diagram of the nasal microbiome based on both studies. The figure shows the unique as well as shared ASVs for the Nose 2.0 and pilot study results. For detailed information on these features, see Supplementary Table 5.

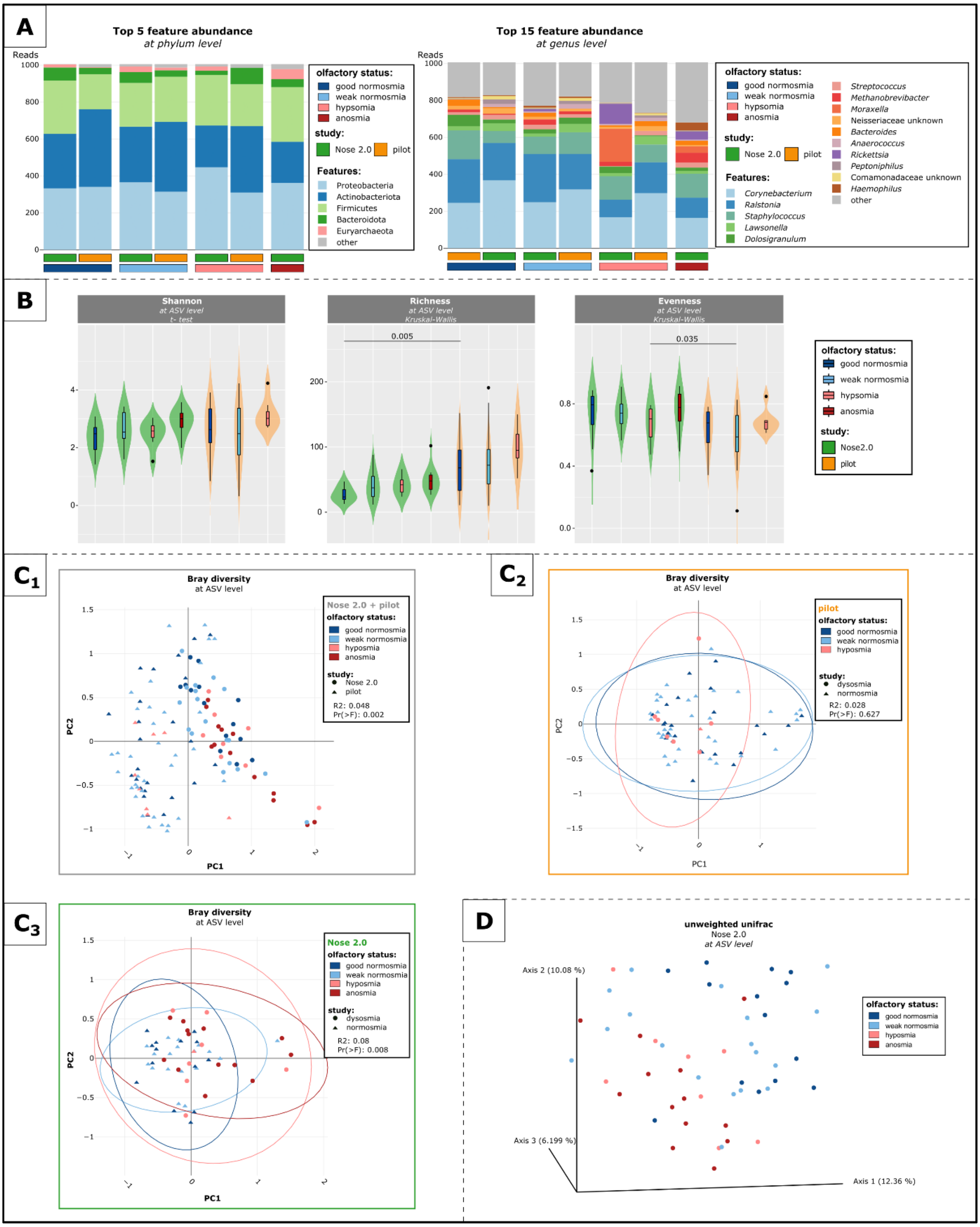

Supplementary Figure 4. Fewer differences between olfactory groups and cohorts after division into olfactory subgroups. **A)** Bar chart of most abundant taxa at the phylum and genus levels. **B)** Alpha diversity indices tended to change stepwise from good to bad olfactory capability. **C)** Beta diversity did not show a shift of the clusters from good to bad olfactory performance as shown in **C1)** the combined (grey box) as well as in **C2 + C3)** each of the studies (orange - pilot; green - Nose2.0). **D)** Unweighted unifrac plot of the Nose 2.0 dataset at ASV level (based on TDI).

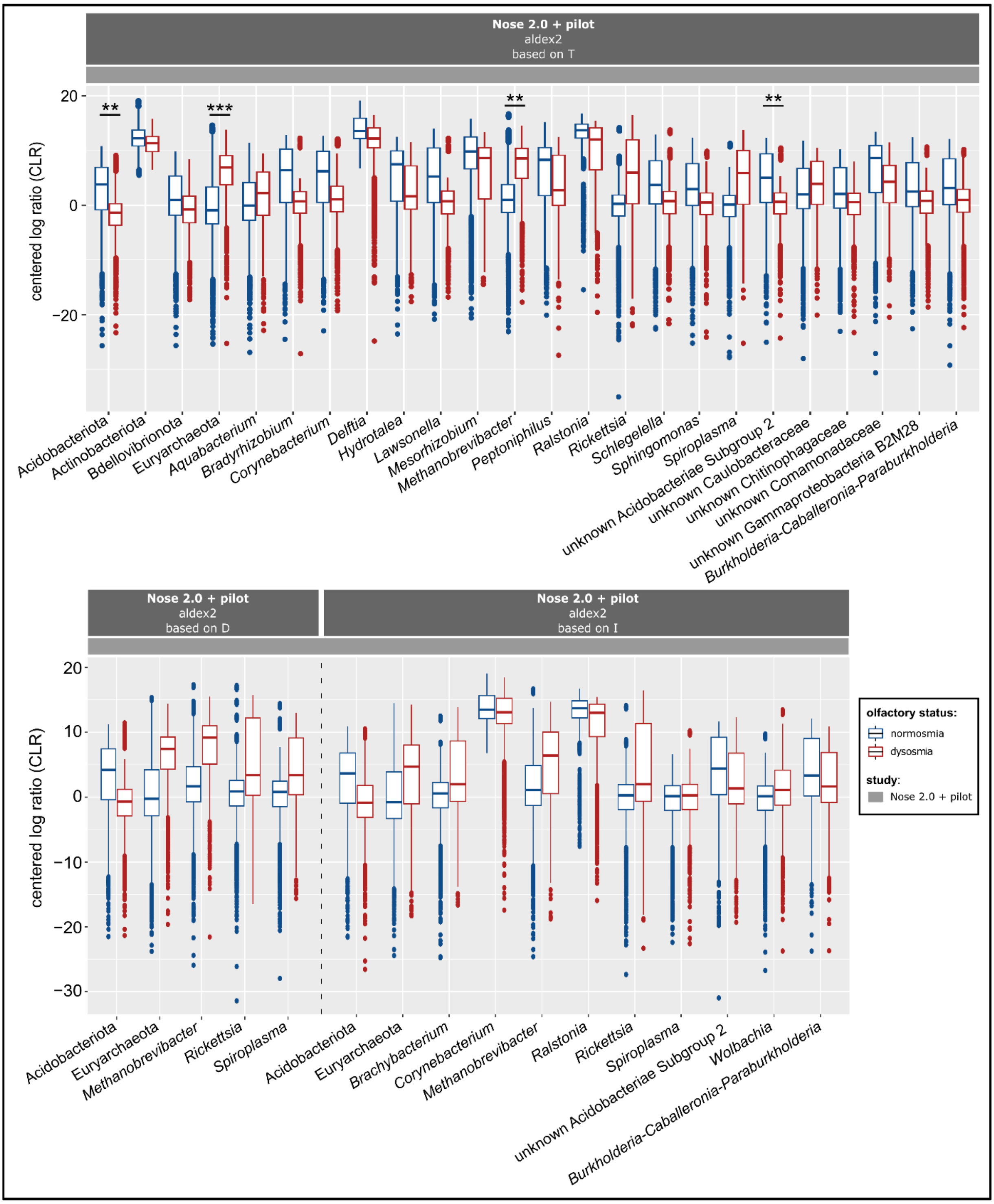

Supplementary Figure 5. Putative microbial biomarkers were found for the olfactory main groups. CLR-transformed data were used to calculate significant differences between the olfactory main groups via ALEDx2. (*q*-values; Kruskal-Wallis test)

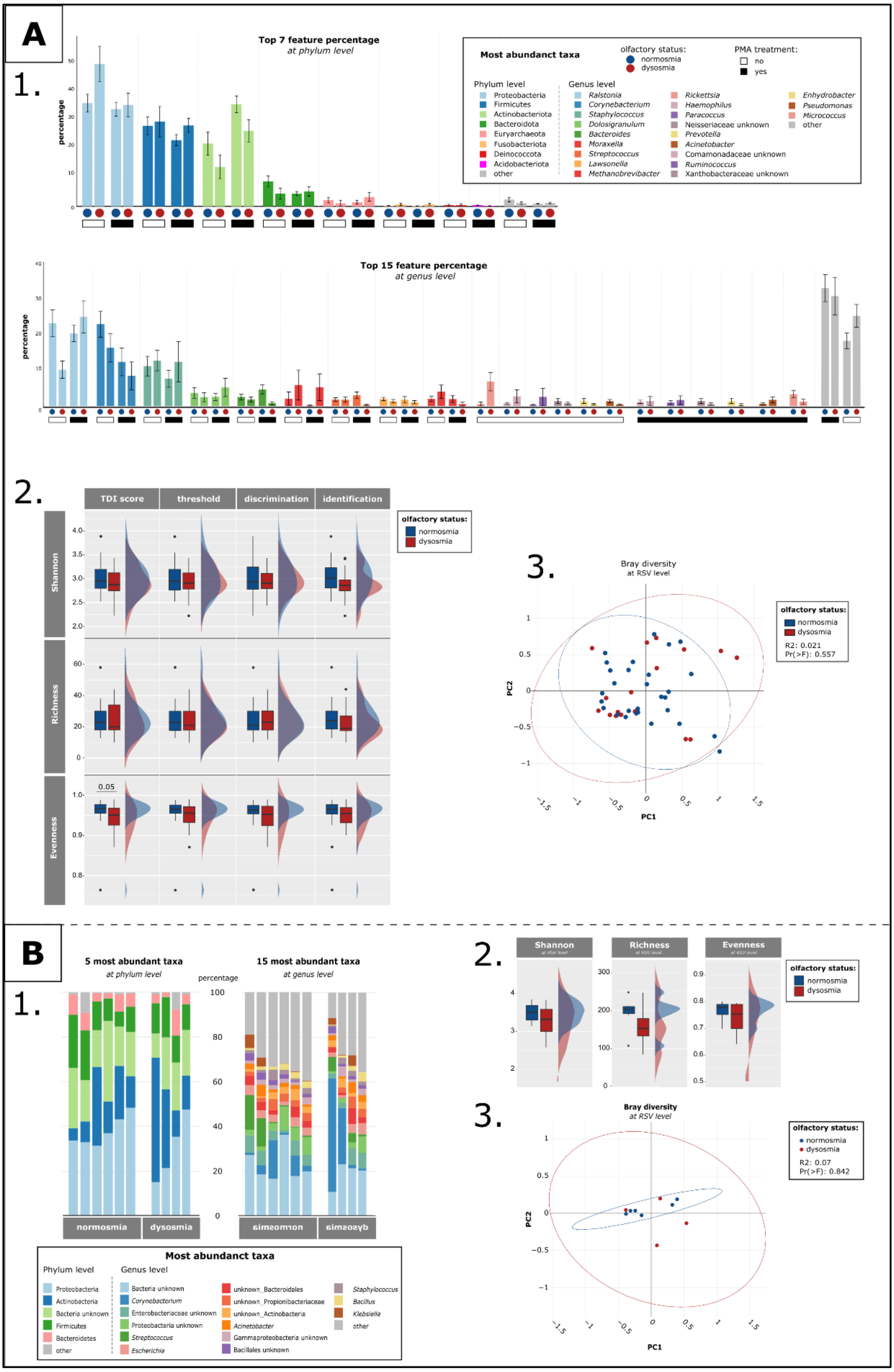

Supplementary Figure 6. Intact cell signatures in the nasal samples show opposite trends compared to overall microbial signatures. This plot shows the **A)** amplicon sequencing results of the propidium monoazide (PMA)-treated nasal samples and **B)** the bacterial output of the metatranscriptomics analysis from the untreated nasal samples. Each of these shows the following: **1.** Most abundant taxa at the phylum and genus levels. **2.** Alpha diversity at the ASV level. **3.** PCA plot at the ASV level. All analyses are based on TDI scores (*p*-values not corrected).

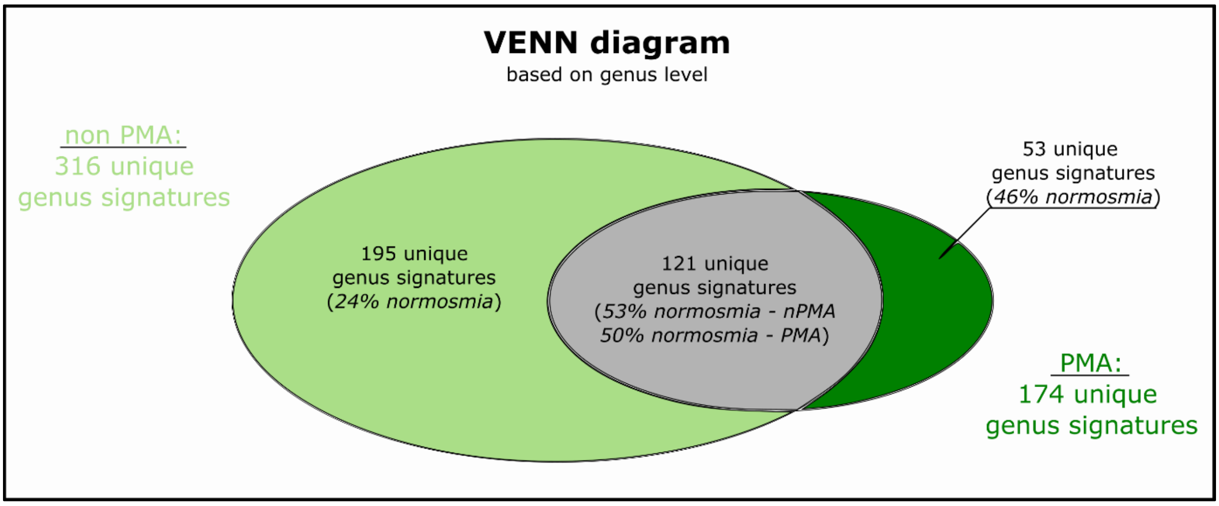

Supplementary Figure 7. Approximately 50% of the shared signatures (between PMA-treated and -untreated) belonged to normosmics. Analysis based on Nose 2.0 only.

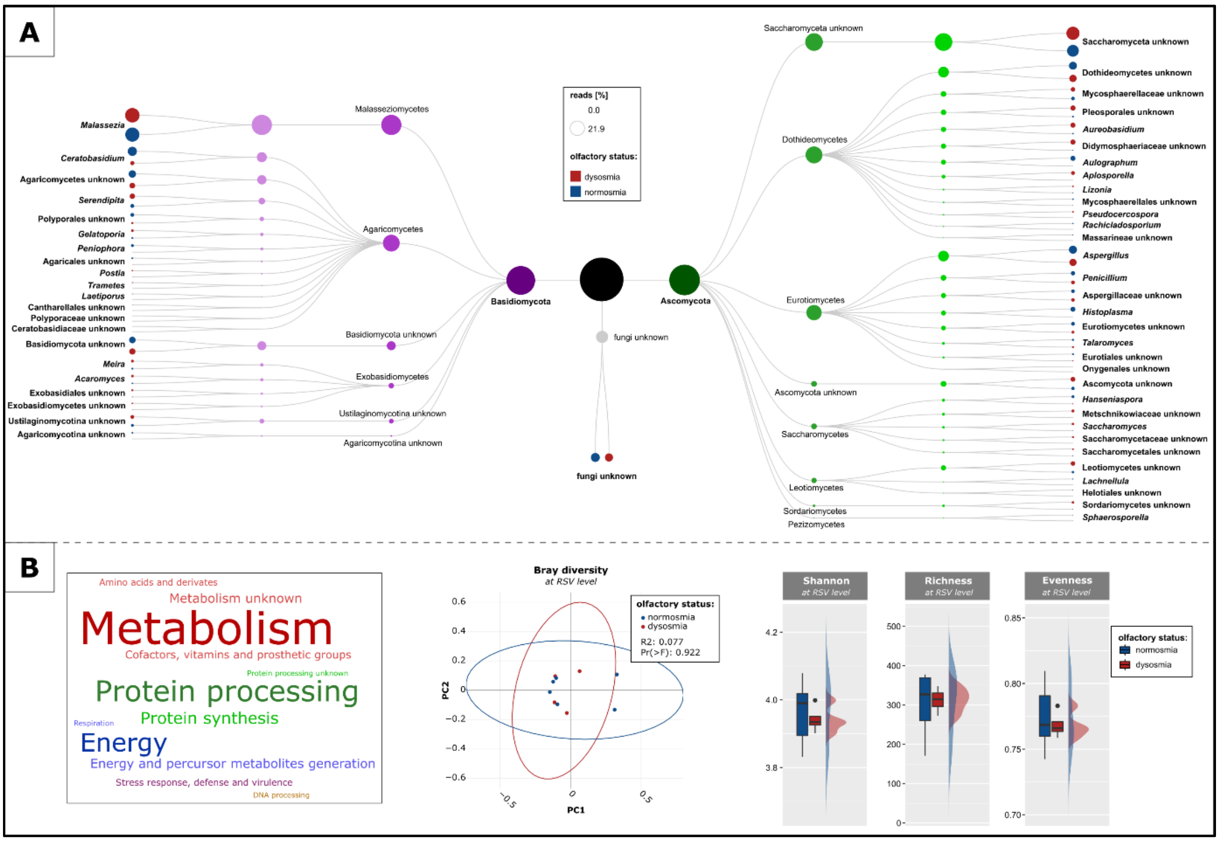

Supplementary Figure 8. No significant differences were observed in fungal community or bacterial functions of nasal samples. **A)** Fungal community (colored by SEED level 1) **B)** Overview of the most abundant bacterial functions and insights into the diversity.

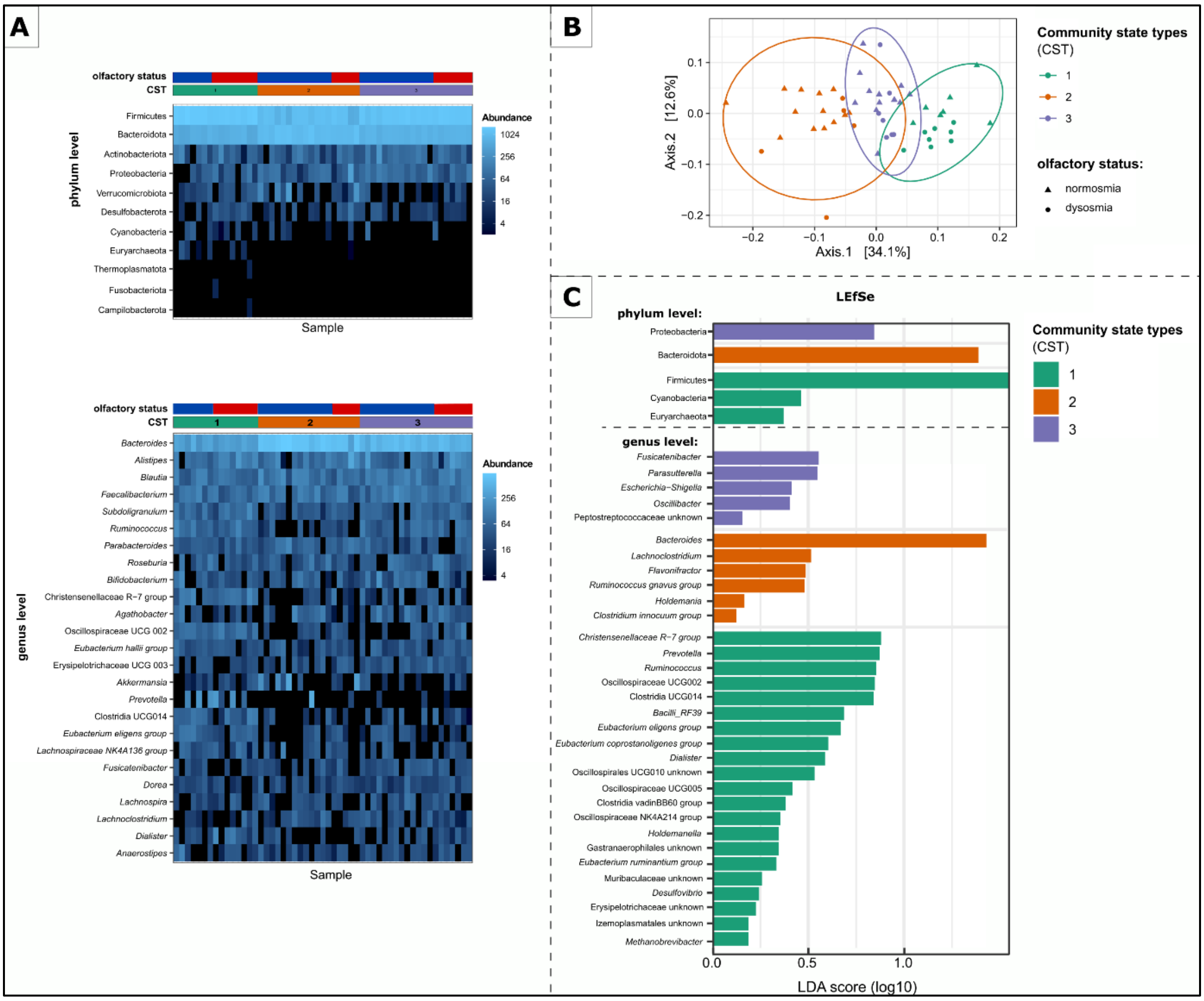

Supplementary Figure 9. Community state types (CST) do not explain differences in stool microbiome based on olfaction. **A)** Associations among the top 11 and 25 abundant taxa at the phylum and genus levels. **B)** Three not-well-separated clusters of the predicted CSTs. **C)** LEfSe showing the biomarkers found for the three community state types. Analysis performed on Nose 2.0 only.

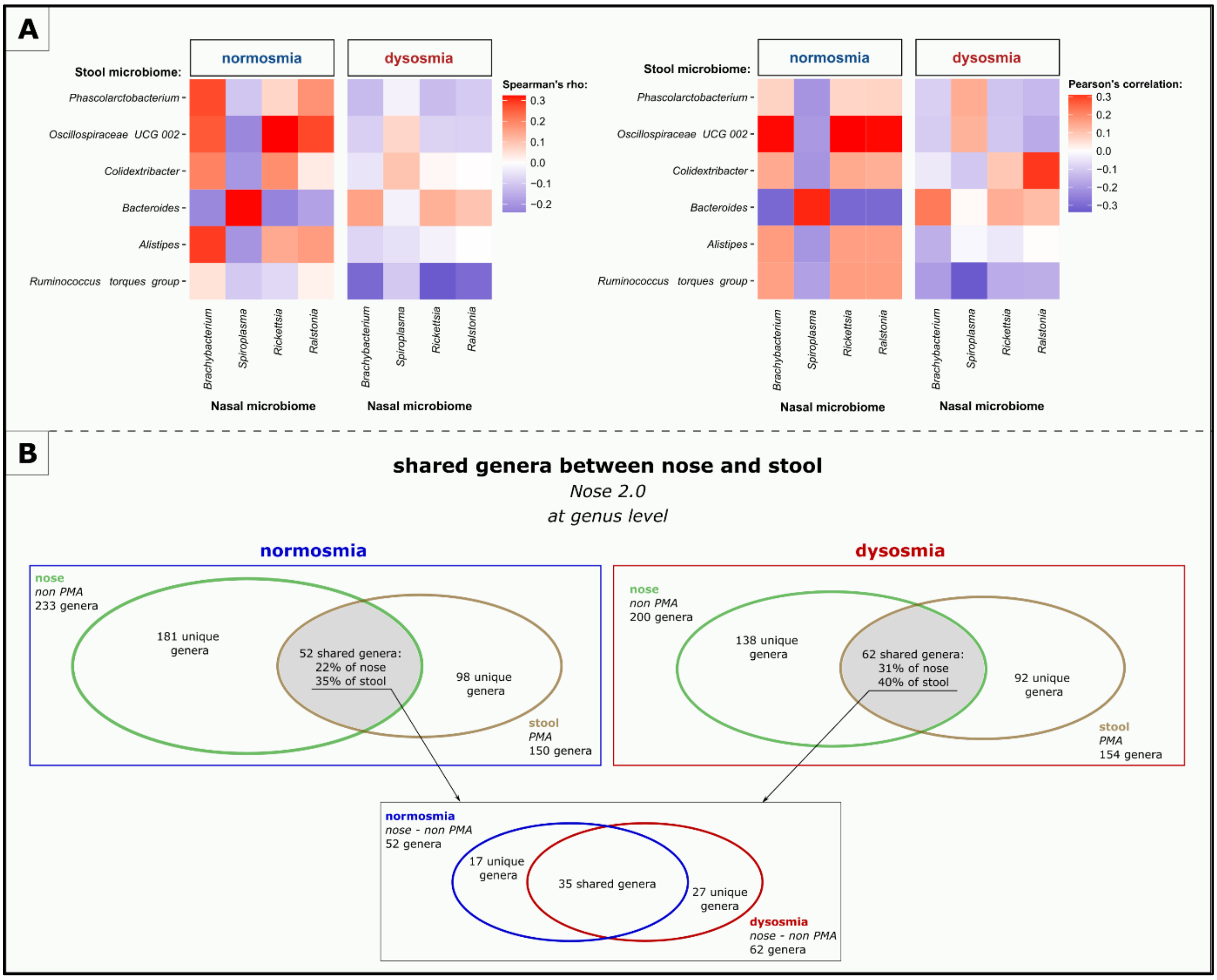

Supplementary Figure 10. No significant correlations were observed between stool and nasal samples. **A)** Correlations between taxa found in nasal and stool samples of normosmics and dysosmics, respectively. **B)** Venn diagram showing the genera shared between nose and stool samples (see also Supplementary Table 13). Analysis based on Nose 2.0 only.

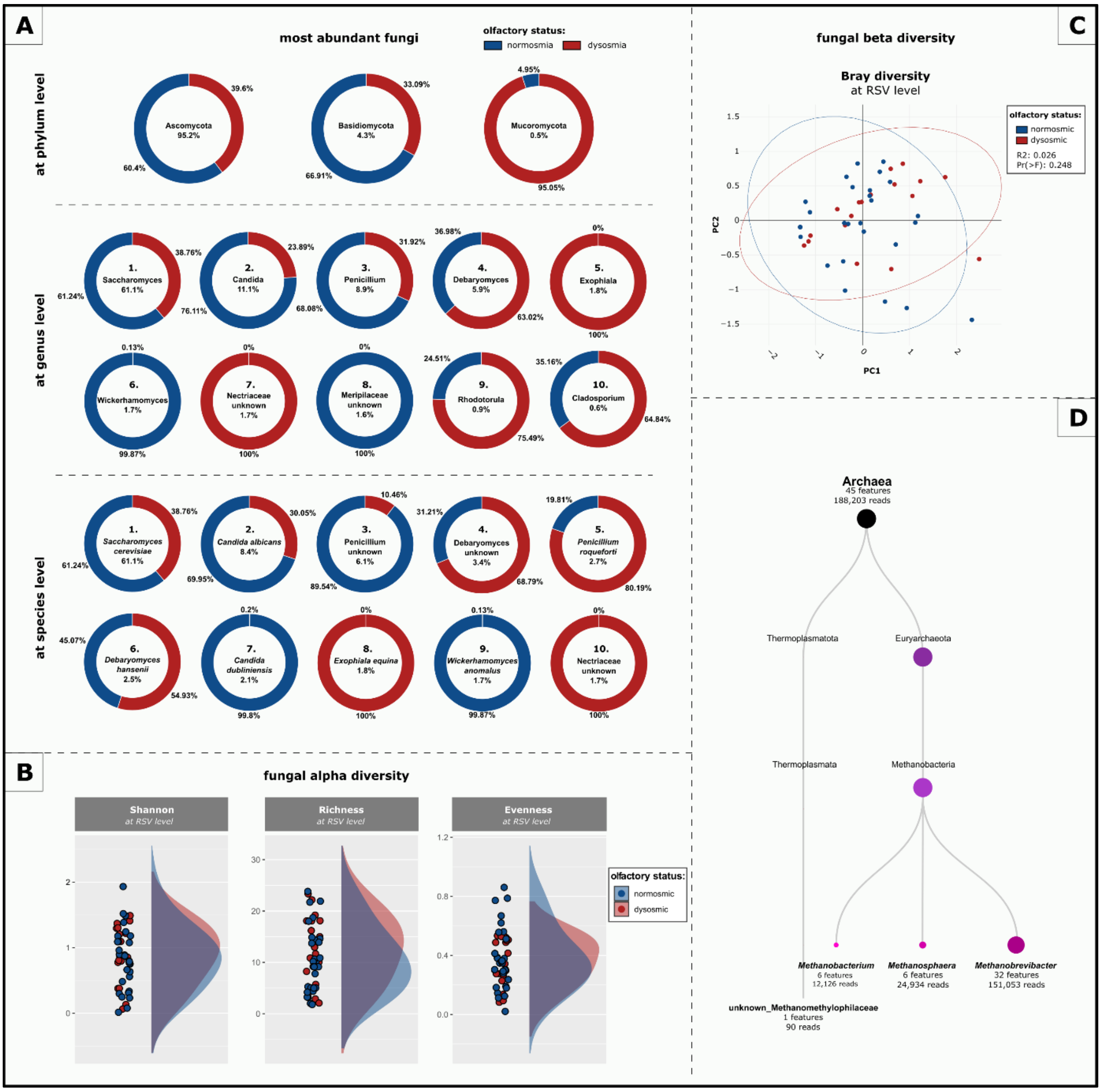

Supplementary Figure 11. Non-bacterial signatures in nasal samples. **A)** Bar chart (and percentages) of the most abundant fungal taxa found in normosmics and dysosmics at different levels. **B)** Alpha diversity indices were similar when comparing the olfactory groups. **C)** Normosmic and dysosmic clusters overlapped in a PCA plot. **D)** All archaeal signatures found in the Nose 2.0 data. Analysis based on Nose 2.0 only.

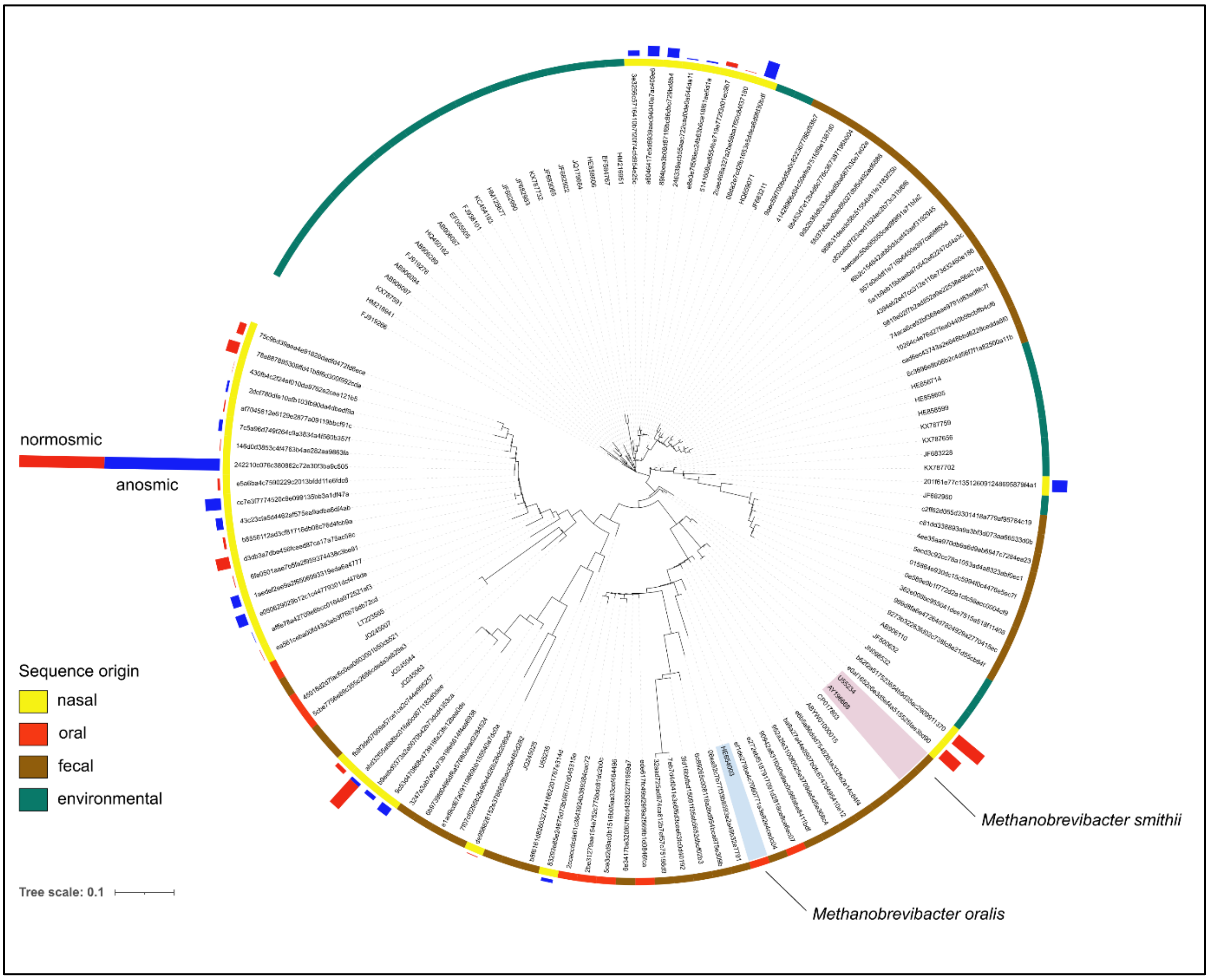

Supplementary Figure 12. Methanobrevibacter signatures from nasal samples in the context of oral, fecal, and environmental Methanobrevibacter signatures. Oral and fecal sequences were obtained from healthy individuals (unpublished data). Additional sequences (e.g., representatives of *M. oralis* and *M. smithii*), as well as environmental sources (rumen), were added to expand the tree. The origin of the sequences is shown in the outer ring, and the absolute abundance (sequence reads) of the nasal *Methanobrevibacter* sequences is displayed as a stacked bar. The most abundant nasal *Methanobrevibacter* sequences cluster separately (left), indicating a separate clade of respiratory-tract-associated *Methanobrevibacter* in both anosmics and normosmics. However, some *Methanobrevibacter* signatures obtained from anosmics clustered with archaeal sequences from fecal samples (right and elsewhere), indicating a potential overlap. Methanobrevibacter signatures obtained from this study. (blue bars represent normosmics' samples, red bars dysosmics' samples)

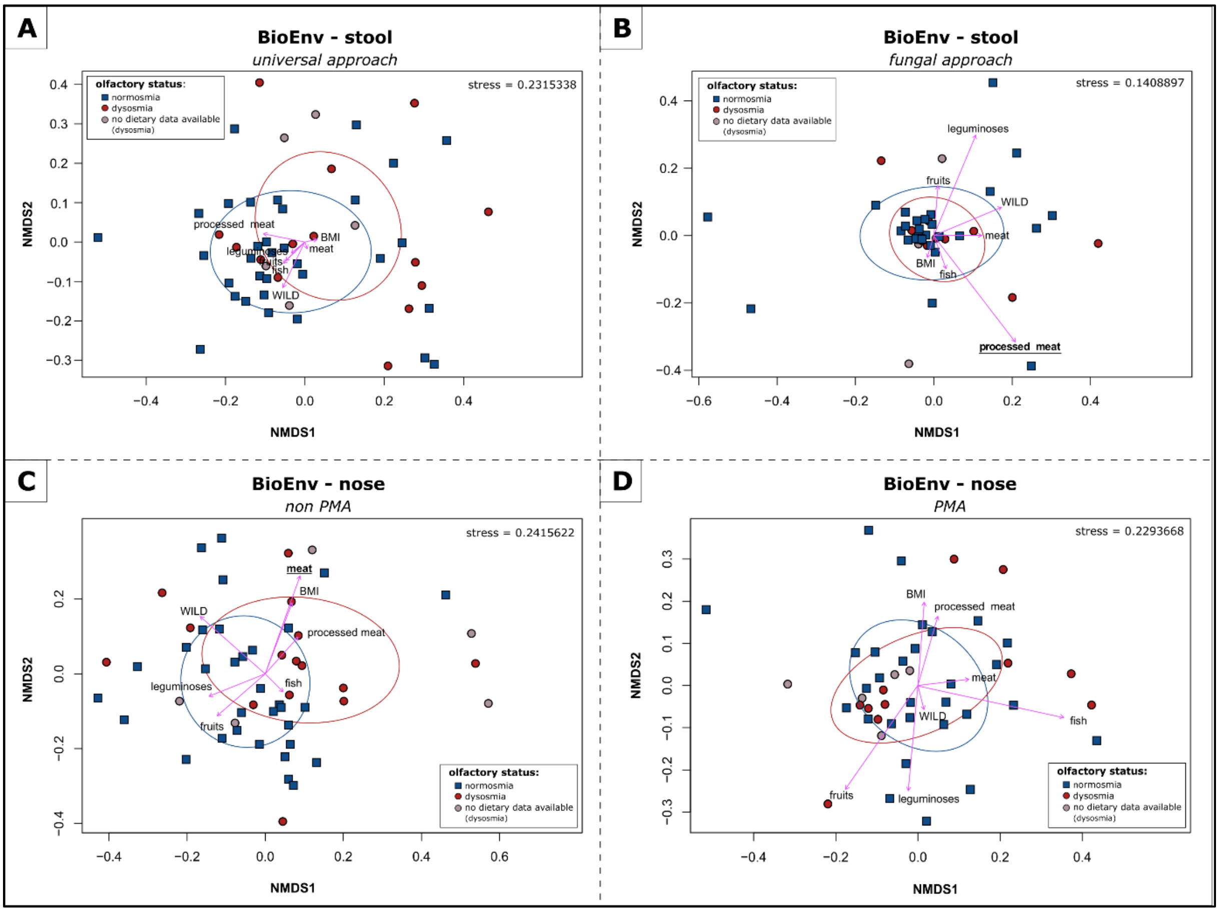

Supplementary Figure 13. The dysosmics' microbiome tended to be correlated with a meat-based diet. The BioEnvs analysis revealed a correlation between dietary information and taxa; on the one hand, this was found by analyzing stool samples using the **A)** universal approach or the **B)** fungal approach, and, on the other hand, this was found in **C)** non-PMA and **D)** PMA nasal samples. Analysis based on Nose 2.0 only.

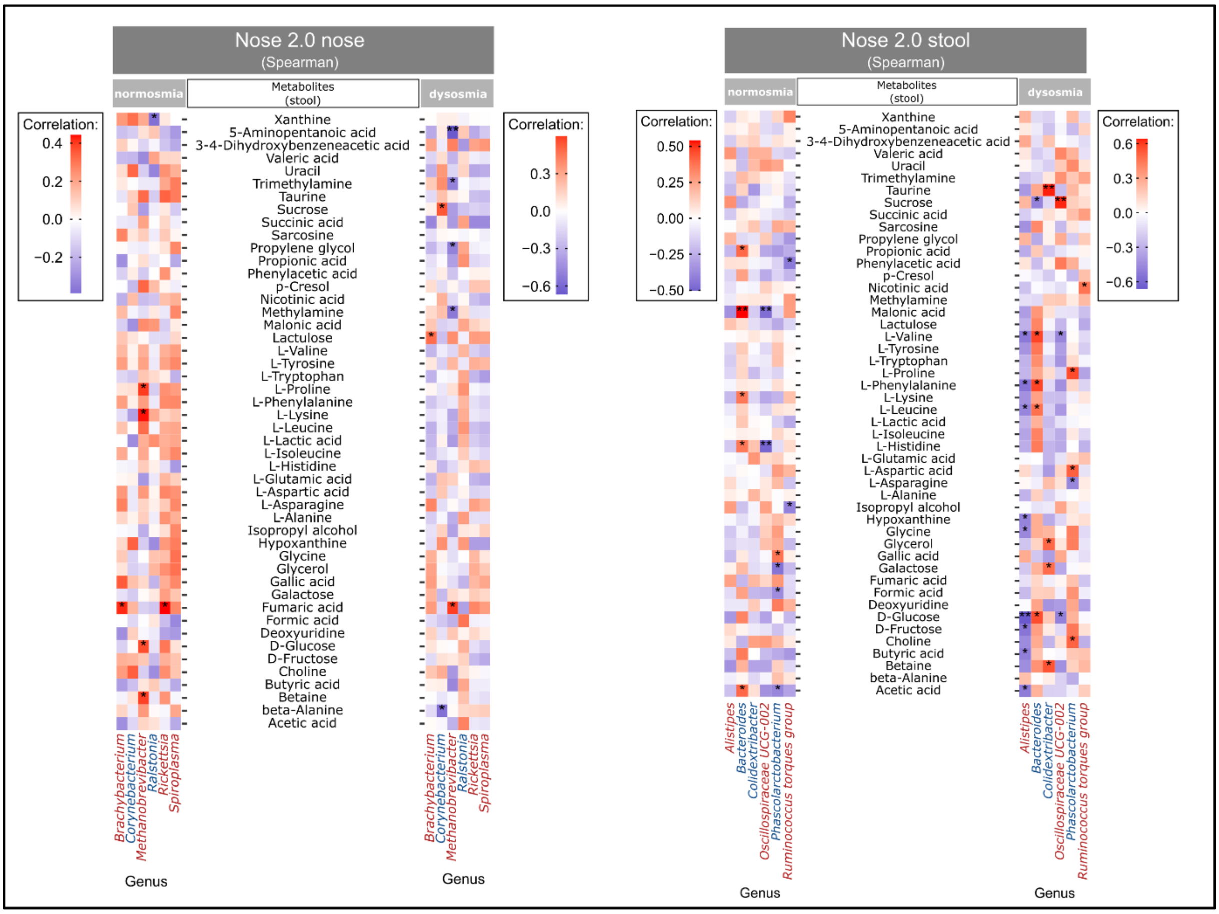

Supplementary Figure 14. Key taxa were associated with several stool metabolites. Heatmaps showing the correlations among metabolites found in stool samples and key taxa in **A)** stool samples and **B)** nasal samples, respectively (*p*-values not corrected; only key taxa of nasal and stool samples are shown). Analysis based on Nose 2.0 only.
