# Supplementary material for: Reduced olfactory performance is associated with changed microbial diversity, oralization, and accumulation of dead biomaterial in the nasal olfactory area
Source: Microbiol Spectr. 2024 Jan 9;12(2):e01549-23. doi: 10.1128/spectrum.01549-23 (PMC10846256; doi:10.1128/spectrum.01549-23)
Supplement: Supplemental text — Commands and settings for data pre-processing (amplicon). [file spectrum.01549-23-s0002.pdf]

Supplementary Information

Reduced olfactory performance is associated with changed microbial diversity, oralization and accumulation of dead biomaterial in the nasal olfactory area

Christina Kumpitsch[1], Florian Ph. S. Fischmeister[2,3,6], Sonja Lackner[4], Sandra Holasek[4], Tobias Madl[5,6], Hansjörg Habisch[5], Axel Wolf[7], Veronika Schöpf[3], Christine Moissl-Eichinger[1,6]

[1] Diagnostic and Research Institute of Hygiene, Microbiology and Environmental Medicine, Medical University of Graz, Graz 8010, Austria

[2] Department of Psychology, University of Graz, Graz 8010, Austria

[3] Department of Biomedical Imaging and Image-guided Therapy, Medical University of Vienna, Vienna, Austria

[4] Otto Loewi Research Center, Division of Immunology, Medical University of Graz, Graz 8010, Austria

[5] Gottfried Schatz Research Center for Cell Signaling, Metabolism and Ageing, Molecular Biology and Biochemistry, Research Unit Integrative Structural Biology, Medical University of Graz, 8010 Graz, Austria

[6] BioTechMed, Graz, Graz 8010, Austria

[7] Department of Otorhinolaryngology, Medical University of Graz, Graz, Austria

Data pre-processing:

1. Load the environment:

QIIME2 v2021.4

2. Import the data:

srun qiime tools import --type 'SampleData[PairedEndSequencesWithQuality]' --input-path *manifest.txt* --output-path *pe-demux.qza* --input-format PairedEndFastqManifestPhred33V2

3. Visualize and check the fastq read quality:

srun qiime demux summarize --i-data *pe-demux.qza* --o-visualization *pe-demux.qzv*

4. DADA2:

srun qiime dada2 denoise-paired --i-demultiplexed-seqs *pe-demux.qza* --p-trunc-len-f XX --p-trunc-len-r XX --p-trim-left-f XX --p-trim-left-r XX --p-n-threads 12 --o-representative-sequences *pe_rep-seqs-dada2.qza* --o-table *pe_table-dada2.qza* --o-denoising-stats *denoising-stats.qza*

DADA2 trunc and trim settings:

	NOSE nonPMA (universal)	NOSE PMA (universal)	STOOL PMA (universal)	STOOL PMA (archaea)	STOOL PMA (fungi)
--p-trunc-len-f	205	205	180	180	220
--p-trunc-len-r	160	160	160	160	160
--p-trim-left-f	10	10			10
--p-trim-left-r	10	10			10

5. Assign taxonomy to your representative sequences

srun qiime feature-classifier classify-sklearn --i-classifier Silva138_16S_99_515F-806R_classifier_V2021.4.qza --i-reads *rep-seqs-dada2.qza* --p-n-jobs 4 --o-classification *taxonomy.qza*

Used classifiers:

universal	Silva138_16S_99_515F-806R_classifier_V2021.4.qza
archaea	Silva138_16S_99_519F-806R_classifier_V2021.4.qza
fungi	unite-ver8.3-99-classifier_QIIME2020.11.qza

Get rid of contaminants using the R package decontam:

1. load libraries

```
library(phyloseq)

library(ggplot2)

library(decontam)
```

2. import your files

```
FeatureTable <- read.table("RSVtable.txt", sep = "\t", header = TRUE, check.names = FALSE, row.names = 1)

metadata <- read.table("metadata.txt", sep = ",", header = TRUE, row.names = 1, check.names = FALSE)

ps <- phyloseq(otu_table(FeatureTable, taxa_are_rows = TRUE), sample_data(metadata))
```

3. remove contaminants

```
sample_data(ps)$is.neg <- sample_data(ps)$sampletype == "control"

contamdf.prev <- isContaminant(ps, method="prevalence", neg="is.neg", threshold=0.5)

table(contamdf.prev$contaminant)
```

4. create a filtered tsv file

```
FeatureTable_clean <- FeatureTable[!contamdf.prev$contaminant, ]

write.table(file = "FeatureTable_clean.tsv", x = data.frame(FeatureTable_clean), sep = "\t", row.names = TRUE, col.names = TRUE)
```
